# GANAB and *N*-Glycans Substrates Are Relevant in Human Physiology, Polycystic Pathology and Multiple Sclerosis: A Review

**DOI:** 10.3390/ijms23137373

**Published:** 2022-07-01

**Authors:** Roberto De Masi, Stefania Orlando

**Affiliations:** 1 Complex Operative Unit of Neurology, “F. Ferrari” Hospital, Casarano, 73042 Lecce, Italy; dmsrrt@gmail.com; 2 Laboratory of Neuroproteomics, Multiple Sclerosis Centre, “F. Ferrari” Hospital, Casarano, 73042 Lecce, Italy

**Keywords:** Multiple Sclerosis, Polycystic Kidney Disease, Polycystic Liver Disease, ER stress, GANAB, PRKCSH

## Abstract

Glycans are one of the four fundamental macromolecular components of living matter, and they are highly regulated in the cell. Their functions are metabolic, structural and modulatory. In particular, ER resident *N*-glycans participate with the Glc_3_Man_9_GlcNAc_2_ highly conserved sequence, in protein folding process, where the physiological balance between glycosylation/deglycosylation on the innermost glucose residue takes place, according GANAB/UGGT concentration ratio. However, under abnormal conditions, the cell adapts to the glucose availability by adopting an aerobic or anaerobic regimen of glycolysis, or to external stimuli through internal or external recognition patterns, so it responds to pathogenic *noxa* with unfolded protein response (UPR). UPR can affect Multiple Sclerosis (MS) and several neurological and metabolic diseases via the BiP stress sensor, resulting in ATF6, PERK and IRE1 activation. Furthermore, the abnormal GANAB expression has been observed in MS, systemic lupus erythematous, male germinal epithelium and predisposed highly replicating cells of the kidney tubules and bile ducts. The latter is the case of Polycystic Liver Disease (PCLD) and Polycystic Kidney Disease (PCKD), where genetically induced GANAB loss affects polycystin-1 (PC1) and polycystin-2 (PC2), resulting in altered protein quality control and cyst formation phenomenon. Our topics resume the role of glycans in cell physiology, highlighting the *N*-glycans one, as a substrate of GANAB, which is an emerging key molecule in MS and other human pathologies.

## 1. Introduction

Glucosidases are a family of ubiquitous intracellular and extracellular hydrolases molecules that normally catalyze the selective hydrolysis of glycosidic covalent linkages on the glycanic substrates.

“Glycan” is a generic term indicating simple and complex carbohydrates or sugars, which represent all carbon hydrates in nature. They express a wide structural complexity of polymerization, ranging from the mono- to oligo- and finally to polysaccharides. However, in all chemical species of glycan a common building block, consisting of [CH_2_O]n as a repeated chemical compound, can be recognized, resulting in a hex-ose or pent-ose ring structural unit. Due to the presence of both carbonyl and alcoholic functional groups in the same molecule, they can cyclize in hemiketals or hemiacetals, which are further classified based on ring size [1]. In fact, five-membered ring systems are designated as furanoses, while the six-membered ones are pyranoses. A part from its D or L chirality, the latter is also known as β- or α-glycoside, depending on whether the glycosidic bond lies “below” or “above” the plane of the cyclic sugar molecule. This also determines the sensitivity to enzymes such as α-amylase, as well as α-glucosidase, only hydrolyzing the α-conformation, or to others, such as emulsin, which affects only the β one. Furthermore, the polar heads forming the [CH_2_O]n element are hydrophilic, conferring to glycans great solubility and metabolic compatibility with the cytosolic and extracellular environment. This is the case of the membrane-bound molecules or many macromolecules subjected to exocytosis and the sialic acid. In fact, according to the possible modifications in its structure at the C-4, C-5, C-7, C-8, and/or C-9 positions, more than 50 different types of sialic acids are known, all constituting the outer cell surface the plasmalemmal glycocalyx [2]. 

Glycans are also highly represented in the soil and nature in general, being able to play both a structural and basal metabolic role in organic matter. The fundamental elements constituting organic matter, which are carbon, hydrogen, oxygen, and nitrogen, collectively make up 99% of the mass of protoplasm. Phosphorus and sulfur are also common elements, essential to the structure of nucleic acids and amino acids, respectively. The interaction degree of these components results in proteins, lipids, nucleic acids, and glycans that in the living system undergo a further stoichiometric enhancement during the combination molecules process, usually generating the catalytic synthesis of glycoconjugates. They comprehend glycolipids and glycoproteins, but also, lipoproteins and essential coenzymes, including Flavin-Adenine-Dinucleotide (FAD) and Nicotinamide-Adenine-Dinucleotide (NAD). Much of this structural variability and complexity is conferred just by glycans, due to their ability to produce polymers and conjugates. In particular, *O*-glycans (*O*-Linked glycans) and *N*-glycans (*N*-Linked glycans) can be covalently linked to the polypeptide via an *N*-acetylgalactosamine (GalNAc) and an OH-group of a serine (Ser) as well as a threonine (Thr) residue or asparagine (Asn), respectively. The resulting structure can be extended into a variety of chemical repetitions, according to the common sequence Asn-X-Ser/Thr, where X is any amino acid except proline (Pro) [3]. However, unlike *N*-glycans having GlcNAc_2_Man_3_ as a common sequence, there is currently no consensus amino acid motif for *O*-glycans known at moment, but only a common structural theme, consisting in the polylactosamine unit. The latter can often be added to the various core structures formed in turn by the repetitive addition of galactose and *N*-acetyl-glucosamine units. Moreover, polylactosamine chains can be capped with the sialic acid, or also with a fucose element, forming a Sialyl Lewis X (sLeX) structure [4].

On the other hand, *N*-glycans can be composed of *N*-acetylgalactosamine, galactose, neuraminic acid, *N*-acetylglucosamine, fucose, mannose, and other monosaccharides. In general, *O*- and *N*-linked glycans are found on the exterior surface of the plasmalemma in eukaryote cells, but they can also be found in prokaryotes, although less commonly. Glycosaminoglycans (GAGs) (or mucopolysaccharides) structurally comprise 2-amino sugars [containing an amino functional group (-NH2) instead of a hydroxylic (-OH) one] linked alternately with a uronic acid (acidic monosaccharide constituted by one or more carboxylic or sulphates groups) resulting in a long negatively charged polymer. All these glycans represent a heterogeneous group of chemical species exhibiting a regulative biological function as well. In fact, since the discovery of DNA, we quickly went beyond the central genetic dogma of “one gene, one protein” to the post-translational analysis of the proteins, their isoforms and maturation. Enzymatic glycosylation represents precisely one of the main post-translational mechanisms and the misfolding checkpoint for the nascent polypeptides. Consequently, the -omics integrative sciences were born to first indicate the complexity of whole genome (genomics), then of the proteins (proteomics), of the lipids (lipidomics) and finally of the glycans (glycomics) [5]. These distinctions are consistent with the complexity of the matter, having reason to exist considering that glycans alone theoretically provide 10^12^ chemical species [6].

The spectrum of all glycan structures is immense; in humans, this is several orders of magnitude greater than the number of proteins encoded by the genome. More than 1% of the human genome encodes proteins that catalyze, localize or bind to sugar chains [7]. Given this large diversity of oligosaccharide structures and the many possible attachment points to most proteins, it is often impossible to foresee and classify glycans. Thus, most study approaches are based on the linkage specificity of enzymes cleaving oligosaccharides, coupled to the HPLC, MALDI-TOF or other mass spectrometric techniques, to obtain a glycan-specific chemical fingerprint. Despite this huge variability and diversity, the key enzymes regulating glycidic branching and metabolism comprehend few molecule families, including mainly glycosyltransferases and glucosidases. Glucosidase II belongs to the latter, which also represents the field of interest in the present work. Specifically, this is an α-glycosidase, known to be in the pathway of the highly conserved normal protein-folding machinery, maintained in the cytosol of both prokaryotic and eukaryotic organisms as well as in eukaryotic mitochondria. In this machinery, *N*-linked glycosylation begins in the endoplasmic reticulum (ER) with subsequent glycan processing during the transport to the Golgi apparatus. Within the Golgi, the interplaying between the glycosidase-mediated mannose trimming and monosaccharide additions via glycosyltransferases, generates an oligomannose chain able to modulate the subsequent protein folding and apparatus homeostasis. In addition, Glucosidase II is involved in human polycystic disease [8,9,10] and finally in the demyelinating ones of the central nervous system (CNS), according to the latest evidence [11]. 

As far as is known, this molecule exerts, due to the metabolic control on its substrates, a wide physiological and pathological influence spectrum, that is uncommon for other similar enzymes, thus arousing our interest. Notably, the knowledge on glycomics is currently scattered in various publications and databases, resulting in a lack of a holistic and systematic view of this matter. In 2012, the U.S. National Research Council called glycoscience a new focus concerning the structures and functions of glycans, also promising great advances in wide areas ranging from medicine to energy generation and materials science [12]. 

In this review, we discuss fundamental concepts in glycobiology, integrating this context with the recent advances in understanding the key roles of the glucosidase II in human health and disease. 

## 2. Glycobiology Overview

We can consider the modern history of glycans as beginning in 1902, with the Nobel Prize for chemistry conferred to German Emil Fischer. The reaction taking his name, developed in the period 1893–1895, refers to the formation of a glycoside in the presence of an acid catalyst, by the interaction of an aldose or ketose with an alcohol. In fact, in these experimental conditions, short reaction times usually lead to the synthesis of the furanose ring structural unit, while longer reaction times lead to the pyranose one. We owe the fundamental studies on the carbohydrates structure, as well as the homonym currently used structural formulas, to Fischer. However, the enormous advancement in the field has motivated the birth of *glycobiology*, that is, the study of the structure, biosynthesis, and biology of saccharides. Due to their chemical spread, the latter had an evolutionistic advantage, representing, in the cell wall, a fundamental compound of lipopolysaccharide (LPS) in the GRAM–, of peptidoglycan (PG) in the GRAM+ bacteria, of nucleocapsid in bacteriophages, of surface in the animal parasitic and free-living nematodes (metazoes), of glycocalyx in the uni- or pluri-cellular eukaryotes, in plants and algae and so on to humans. This ubiquitous diffusion of saccharides over the last 2–3 billion years, among living matter, is sustained by their physical properties, depending on molecular weight and bonds between the disaccharide repeating units that facilitate the extreme solubility as well as steric flexibility in the water of the glycans molecules [13]. On the other hand, the absence of a coding template, unlike proteins, is responsible for the immense chemical spread of glycans. 

On that basis, most glycan functions are mediated not by a single absolutely required structural sequence, but by a spectrum of similar structures, working more in “analog” than in *lock-and-key* “digital” manner [13]. However vast, this molecular diversity is limited, and a phenomenon of evolutionistic convergence took place during phylogeny. Thus, sulfation turned out the main dynamic post-translational modification process of glycans. It can occur in various positions within the GAG backbone and modulates extracellular signals such as cell–cell and cell–matrix interactions [14]. GAGs in turn are a common key element of the pericellular space and extracellular matrix, determining the physical characteristics of tissues and modulating biological functions of cells. Among these properties, the negative charge for all GAGs is paramount for their functions, and due to unknown reasons, positively charged glycans are uncommon in nature [15]. 

The same convergence mechanism refers to Asn-X-Ser/Thr co-translationally linked mannose signal Glc_3_Man_9_GlcNAc_2_, a highly conserved oligosaccharide structure. The latter represents the substrate of Glucosidase II, the key enzyme of the proteins’ quality control machinery. In fact, the fine balance between glycosylation and de-glycosylation of substrates modulates many cell structures and biological functions. However, glycans are also involved in other processes and functions—basal metabolism in the case of monosaccharide molecules; energy storage in the case of polysaccharides such as glycogen; protein folding with protein–protein interaction and cell–cell recognition; or the structural role in the case of glycocalyx constituting the eukaryotic cell wall, of PG or LPS in the prokaryotes as well as chitin in the arthropods and cellulose in vegetables. Finally, many molecules, including glycoproteins and glycolipids, are suitable for therapeutic usage in humans.

## 3. The Roles of Glycans

In both unicellular and pluricellular organisms, the basal metabolism works with energy extraction through the degradation of carbohydrates, mainly in the form of glucose. A detailed discussion of this field exceeds the aims of the present work. For a more in-depth study on this matter, please refer to “Carbohydrates metabolism” of Mondal, 2019 [16]. In this section, we will limit ourselves to the notions of topical relevance.

Although widespread in nature, the glycolytic pathway shunting from the aerobic to anaerobic conditions, depending on the oxygen concentration, do not apply to cancer cells. The latter, in fact, respond to the Warburg effect [17]. In tumor cells, the rate of glucose uptake dramatically increases, and lactate is produced, even in the presence of oxygen and fully functioning mitochondria. In this case, the upregulated M2 isoform of Pyruvate kinase has a key-role [18]. This differential metabolic property of cancer cells is also at the basis of modern radiometabolic examinations by positron emission tomography (PET) in discriminating suspected lesions from surrounding normal tissue [19].

On the other hand, gluconeogenesis is the opposing metabolic pathway of glycolysis, but it is not its exact reversal, even though it shares a number of enzymes. The starting substrates of gluconeogenesis are the glycerol, lactate, and α-keto acids. Interestingly, the main sites for the regulation of glycolysis and gluconeogenesis are the phosphofructokinase-1 (PFK-1) and fructose-1, 6-bisphosphatase (F-1, 6-BPase) catalyzed reactions. These enzymes regulate the phosphorylation balance of monosaccharides, between fructose 6-phosphate e fructose 1,6-biphosphate. A large part of gluconeogenic glucose undergoes cytosolic glycogenesis. 

Notably, one-three percent of glycogen is continuously degraded by the lysosomal enzyme, α(1→4)-glucosidase (acid maltase) with an unknown purpose. However, a deficiency of this enzyme causes an accumulation of glycogen in vacuoles in the lysosomes, resulting in the serious glycogen storage disease type II called the Pompe disease [20].

At present, other enzymatic defects of the glycans metabolism are known, resulting in eight glycogen storage diseases (GSD) also called glycogenosis and dextrinosis [21].

Finally, when glucose intake is deficient or insufficient for energy production, increased mobilization of fat from adipose tissue occurs. The fat metabolism is incomplete when glycolysis is lacking, resulting in the production of large amounts of ketone bodies, such us Acetate, Acetoacetate and β-hydroxybutarate. The early phase of this condition is described as physiological for the CNS, being entirely dependent the glycidic metabolism and also able to extract the ATP molecules from ketone bodies; instead, the advanced phase, known as “ketosis”, is characterized by severe acidosis and ultimately by coma [22]. 

The structural role of glycans is derived from their property to polymerize by forming biological barriers and structures. Flexibility is another important parameter that determines the elasticity and the level of interaction with the surrounding environment. Linked homopolymers of glucose for cellulose and *N*-acetylglucosamine for chitin are the most abundant organic molecules on the planet, providing structures such as plant and fungal cell walls and arthropod exoskeletons [23,24]. These polymers tend to be rigid and resistant to physical, chemical or enzymatic agents. In contrast, mucins appear as a dense fluid layer coating many epithelial surfaces of airways and intestines, providing a critical barrier against microorganisms [25,26]. In fact, glycosylated secretions produced in large amounts can serve to physically expel bacterial intruders. However, a concomitant lubrication effect is also described for mucins in these anatomical sites. This is the case of the muciparous goblet cells of the respiratory or intestinal epithelium [27]. Solubility in the bloodstream of unbound molecules is the main effect of glycosylation, without which the process would probably be impossible. Coherently, the serum protein concentration of ~50–70 mg/mL in humans implies a remarkable ~2 mM of linked sialic acids [13]. The antifreeze function, which prevents the formation of ice crystals in body fluids, is also described in some fish [28]. This property is mediated by polysaccharides with a lipid component [29]. In vertebrates, many components of the extracellular matrix are glycan polymers such as sulfated glycosaminoglycans and hyaluronan. These polymers are self-complexed with specific proteins, resulting in larger macromolecules generating structures such as basement membranes [30,31] and cartilage [32,33,34]. Finally, emerging observations focused on glycans in the biofilms of bacterial multicellular communities with new perspectives in antibiotics discovery [35]. 

Finally, glycans can have profound effects also on the organization of cell membranes and glycocalyx. Although a detailed discussion of this field exceeds the aims of the present work, some critical modulatory roles of glycans need to be highlighted as appropriate. About this, the ability of glycans to form barriers does not disregard a modulatory role on them. The structural flexibility and low-force interaction of the intrinsic negative charge with the extracellular matrix (ECM) components underlie this modulatory property and confer to them a quality of “plasticity” [36]. Bulky negative charged glycoproteins of glycocalyx can modulate cell–cell adhesion and cell–matrix interactions also by applying tension to matrix-bound molecules, resulting in integrin activation and clustering [36]. On these bases, both the adhesive and the anti-adhesive actions of glycan have been described. In fact, due to both bulk and negative charges, hyaluronan and polysialic acid can inhibit cell–cell adhesion and cell–matrix interaction [37,38]. Cell surface glycoproteins can also modulate the membrane domain organization in this way. This is the case of GPI-anchored proteins mainly associated with glycolipid-enriched membrane microdomains interacting with lectins [39,40] or in the case of sialylated ganglioside GM3, which interacts with tyrosine kinase signaling of EGFR and insulin receptors [41,42,43]. Some observations suggest the existence of the self-organizing lectin-based lattices linked to branched glycans [44,45,46]. These ordered structures within the glycocalyx are thought to alter interactions between cell surface molecules, until affecting their membrane trafficking by endocytosis. 

Furthermore, it is known that various degrees of branching in the *N*-linked glycans of surface glycoproteins can affect their functions [47]. This also refers to the regulation of cytokine receptors and modulation of endocytosis rates, resulting in the control of cell proliferation and differentiation, as well as clearance from the circulation [48]. Glycosaminoglycans can be so thick on the cell surface that they form growth factor binding gradients [49]. 

This particular bulking effect induces, in turn, the morphogen gradient during the developmental phase [50,51,52,53]. The acrosome reaction itself is known to be a glycocalyx-mediated process. Specifically, *N*-Glycolylneuraminic acid (Neu5Gc) is a sialic acid, a lack of which, in males, leads to higher fertilization rates, and, on the contrary, to lower rates in females [54]. Moreover, the bulking effect of glycans is responsible for biological masking or protection, avoiding the recognition of the underlying glycan by specific glycan-binding proteins [55]. In fact, *O*-acetyl modifications of terminal sialic acid can block the binding of some influenza viruses [55,56].

Another example is the sulfate-mediated extracellular removal of binding sites for heparan sulfate ligands, resulting in modifications of the interferon (IFN)-beta/IFNAR signaling pathway [57,58,59,60]. Glycan branching is also known to modulate malignant transformation and T-cell activation [61,62]. However, larger glycans typically disrupt peptide loading on T-cell receptors (TCR) during the conjunction with the antigen processing cell [63], representing a common immune escape strategy of highly glycosylated enveloped viruses [64,65]. This is a well-known mechanism of protection from immune recognition that generates immunotolerance. To this end, an unusual pentasaccharide repeat called polysaccharide A, derived from mammalian gut microbiome, is known to modulate the host immune system by inducing a tolerant state through the engagement of T-reg [66]. The control of diffusion barriers is another property of glycans, which contributes to the modulation of the permeability and physical composition of the cell wall.

Main examples of this property are represented by podocalyxin on glomerular podocyte [67,68,69] and heparan sulfate glycosaminoglycans on the glomerular basement membrane [70,71]. These structures are thought to be important in maintaining the integrity of blood plasma filtration by the kidney.

More recently, the adhesion, tethering and rolling of lymphocytes on the blood–brain barrier (BBB) during the early phase of CNS invasion are also recognized as mediated by glycoproteins in Multiple Sclerosis (MS) and related disorders [72,73]. Thus, the complex signaling pathway is constituted by the sequence of selectins, chemokines and integrins at the interface blood/CNS, resulting in emperipolesis through matrix metalloproteinases type 9 (MMP9) activation and tight-junctions disruption [74,75]. It is now evident that nucleocytoplasmic glycosylation functionally characterizes many proteins according to an allosteric mechanism [76]. Furthermore, the *O*-linked *N*-acetylglucosamine (*O*-GlcNAc) modification can work with or against the Ser/Thr phosphorylation, affecting numerous physiological and pathological processes. Specifically, the size, number, branching and degree of glycan sialylation can generate numerous glycoforms of a single polypeptide, influencing its activity. This is the case with erythropoietin [77,78,79] and granulocyte-macrophage colony-stimulating factor (GM-CSF) [80,81]. It has been shown that IgG against GM-CSF and IFN type I, another glycoprotein, are responsible, respectively, for cryptococcosis and recurrent HSV1 encephalitis in humans [82,83].

Another example of function modulation depending on the structural features of the *N*-glycans is the incomplete galactosylation of the IgG-Fc region that has been associated with chronic inflammatory diseases [84,85,86]. On the contrary, the sialylation of this region appears to confer anti-inflammatory properties exploited in therapeutic usage of intravenous immunoglobulins in humans. Finally, the addition of *O*-GlcNAc residues to histones-binding DNA is a key component of epigenetic modifications that regulate chromatin organization and gene expression. The mechanism involves the *O*-GlcNAc transferase encoded on the X chromosome, resulting in inactivation and genetic imprinting [87,88,89].

## 4. Recognition Patterns of Glycans

According to the aforementioned chemical and physical properties of glycans, it is not surprising that numerous pathogens and symbionts have developed highly specific ways of recognizing glycans on the host cell surface or that highly conserved sequences of specific glycan-binding proteins participate in a wide variety of cell functions. 

Although many researchers claim that the glycan-dependent process is not important, these assumptions are often obtained under static experimental conditions, making the glycan role appear marginal. An exhaustive discussion on the glycans recognition patterns exceeds the aim of the present work, but we believe that it is necessary to cite the most explanatory cases.

Bacterial, fungal and parasite adhesins are known, as well as viral agglutinins. For example, Helicobacter Pylori recognition of gastric sialoglycans is particularly interesting, given its involvement in gastric ulcers and cancers [90,91,92]. Plasmodium falciparum causes malaria through recognition of densely sialylated glycophorins on target erythrocytes [93,94]. Viral glycan-binding proteins (hemagglutinins) such as 9-*O*-acetyl ester on the sialic acid side chain of certain coronaviruses and influenza C and D viruses are critical for binding host cells [56,95,96]. The bacteriophages themselves recognize the surface polysaccharides as a bacterial target. Consistently, the diversity of surface polysaccharides found on some species like pneumococcus can be explained by selection for evasion from the vertebrate antibody response as well as bacteriophages [97]. In fact, noroviruses can be affected in their infection spreading as they selectively bind to one blood group of ABO structure and not another [98,99]. This results in a host escape mechanism. In other instances, pathogen glycosidases represent a virulence factor. This refers to the flu virus and vibrio cholerae [100]. 

As for the influenza virus, its sialic acid-binding (the hemagglutinating, H) activity is balanced by the sialic acid-releasing enzyme (the neuraminidase, N) resulting in the cleavage of interfering molecules and accessibility of viruses to the cell surface [101,102]. Not by chance, the specific neuraminidase inhibitor zanamivir (Relenza) [103,104] projected onto the previously known sialidase inhibitor Neu5Ac2en [105] is the approved drug for preventing the human infection. Similarly, the neuraminidase of vibrio cholerae confers virulence by removing all but one specific residue of sialic acid from host surface gangliosides, the GM1 monosialoganglioside. The latter is the specific receptor for the B subunit of the AB5 choleric exotoxin [106].

In immunological terms, it is also known that immune cells can elicit the innate immune response by detecting damage-associated molecular patterns (DAMPs) or pathogen-associated molecular patterns (PAMPs) using Pattern Recognition Receptors (PRRs) [107], such as Toll-like receptors (TLRs) [108,109], NOD-like receptors (NLRs) [110,111] and C-type lectins [112,113]. Many PAMPs or DAMPs are made up of glycoconjugates such as LPS, PGs and RNA and DNA-derived (deoxy)ribose-based polymers [114]. This evidence is even more important considering that an adaptive immune response cannot take place without the innate one first. Glycans are also known to work as self-associated molecular patterns (SAMPs) [115], being recognized by intrinsic inhibitory receptors to maintain the immune tolerance for self-antigens and to dampen immune response. In particular, surface sialoglycans provide a mechanism to allow the host to discriminate between infectious non-self from non-infectious self [116]. 

The same mechanism of glycan or glycoconjugates recognition pattern underlies the molecular mimicry. Molecular mimicry refers to the pathogenesis of many human pathologies such as demyelinating diseases such as MS [117] and Neuromyelitis Optica/Neuromyelitis Optica Spectrum Disorders (NMO/NMOSD) [118,119], Bickerstaff’s brainstem encephalitis (BBE) [120], chronic inflammatory disease polyneuropathy (CIDP) [121] and other organ-specific human chronic inflammatory diseases as well as the acute polyneuropathy and Guillain–Barré–Strohl syndrome (GBS) [122]. In all these conditions, a hypersensitivity process induces tissue damage during cross-reaction against a foreign self-antigen. Known glycan self-antigens are MOG for MS, Myelin oligodendrocyte glycoprotein antibody-associated disease (MOGAD) and NMO/NMOSD [123,124,125]; MAG for human demyelinating neuropathy [126]; GM1, GD1a, GT1a and GQ1b for GBS [127]; the latter also for BBE [128]; and *N*-acetylglucosamine-6-sulfatase (GNS) for Rheumatoid Arthritis [129]. 

Like molecular mimicry, some microorganisms expressing endogenous glycans can escape the host defense immune reaction. Consequently, because glycans are often targets of many infectious agents, intra- and interspecies polymorphisms in the cross-reaction on such targets can provide herd immunity, resulting in limited disease spread. Finally, the presence of high densities of terminal Man or GlcNAc residues on foreign proteins or microbes can trigger their phagocytosis via C-type lectins on antigen presenting cells [130,131].

Intrinsic recognition patterns of glycans substantially refer to intracellular protein folding, degradation and trafficking as well as triggering of endocytosis and phagocytosis.

Intracellular protein folding and degradation comprehends a complex pathway from nascent protein regulation to ER-associated degradation (ERAD). In fact, in 1978 Li and coworkers fully described the unusual Glc_3_Man_9_GlcNAc_2_-P-P-dolichol as the highly conserved glycan sequence of lipid-linked oligosaccharide donor for *N*-glycosylation of nascent proteins [132]. Based on the presence of mannose 6-phophate, the enzymatic array of glycan-modifying and glycan-recognizing proteins determines the fate of a glycoprotein molecule in the ER—whether it will be allowed to go from the ER into the Golgi machinery as a final destination, or be consigned for ERAD [133,134]. Specifically, *O*-mannosylation and *O*-fucosylation can monitor the folding of newly synthesized proteins by removing them in case of failed folding, via reverse translocation into the cytosol and finally going to the proteasome after ubiquitination (see below). The mannose 6-phosphate recognition system for the targeting of unfolded proteins to lysosomes is also the classic example of intracellular trafficking of specific glycoproteins involved in triggering endocytosis and phagocytosis. However, there is also evidence for other lectin-like molecules, which are thought to be involved in the ER–Golgi pathway [135].

## 5. Glycosylation in Health and Disease

Glycosylation or deglycosylation is an important topic in cell physiology because gly-cans contribute to protein functions through their alternative glycosylation. In fact, the ad-dition of different glycans on the same polypeptide attachment site can modulate its properties. Glycans express diversified chemical species and functions based on the gly-cosylation site and the quality of the involved compound, resulting in different types of gly-cosylation. In particular, the latter can be characterized by macroheterogeneity, referring to the site occupancy or completeness of glycosylation, while microheterogeneity concerns variations of glycan structure in a specific site compound [136,137]. Consistently, the degree, type and heterogeneity of glycosylation significantly impact physical and biochemical properties of proteins, and they are critical for normal physiology and disease development.

### 5.1. The Enzymatic Glycosylation and Its Modulation

The enzymatic glycosylation is a form of post-translational modification of a polypeptide chain, lipid, polynucleotide, carbohydrate, or other organic compound by the enzyme-catalyzed covalent attachment of carbohydrate from a donor substrate, generally catalyzed by glycosyltransferases in the ER. On the other hand, glycoside hydrolases or glycosidases are enzymes breaking glycosidic bonds from organic compounds [138]. The balancing between glycosyltransferases and glycosidases confers a specific degree of glycosylation and the specificity of the compound linked glycides, such as a sugar code. The sugar code (glycocode) is a coding system based on linked carbohydrates that modulate compounds’ functions [139]. This glycanic coding system is template-free, also having a large capacity due to the huge theoretical number of mono-, oligo-, or poly-saccharides in nature (much higher than that formed by nucleotides) [140]. Sequences of glycocode and related phenotypes can occur through genetic or epigenetic variants of glycogenes. These genes can have a pleiotropic effect on glycosylation by acting on the glycosyltransferases/glycosidases balance, influencing, in the end, cell metabolism and functions. Therefore, omics data belonging to system glycomics (DNA methylation, transcriptomic, proteomic, glycomics, etc.) have been shown to improve glycobiology study and comprehension. For example, cytogenetic aberrations with hyper-diploidy, 1q21 gain, and 13q14 deletion have been associated with glycogene expression patterns in multiple myeloma (MM). Among 243 glycogenes, 60 showed a significantly higher expression in MM than normal plasma cell samples, while 20 showed a lower one [141]. Regarding prostate cancer, a net molecular signature suggests a prevalence of glycosylation enzymes with a missense variant rs61752561 resulting in prostate specific antigen (PSA) extra glycosylation as well as other somatic variants causing the potential loss of glycosylation [142]. Moreover, an altered expression of a cancer-associated glycosyltransferase ST6GAL1 has been identified in colorectal cancer (CRC) [143]. Finally, an altered expression of genes controlling core fucosylation has been recognized as responsible for hepatocellular carcinoma [144]. Numerous studies using different methodologies have been published indicating various structural alterations such as sialylation, fucosylation, degree of branching, and the expression of specific glycosyltransferases associated with breast, colon, liver, skin, ovary, bladder cancer, and neurological disorders [145,146,147]. For example, cerebrospinal fluid glycosylation pattern was used for early diagnosis of Alzheimer’s disease [147]. 

Furthermore, most FDA-approved tumors biomarkers currently used in clinical practice are glycoproteins that mainly show an altered glycosylation pattern [148]. The only form of glycosylation found in the mammalian nucleus is the *O*-GlcNAc type, marking histone, suggesting that the glycosylation itself can regulate gene transcription [149]. There are other covalent post-translational modifications of histones including methylation, acetylation and ubiquitylation. These are dynamically regulated by interplaying enzymatic pairs, which add and remove these modifications in a fine balance and regulation [149]. 

Recently, noncoding RNAs have been studied in relation to glycans and several miRNA regulators mapped on glycogenes indentifying glyco-miRNAs. The glycosylation regulated by glyco-miRNAs provides a link between miRNA-mediated control of cell phenotype and cellular glycanic compounds [150]. For example, long intervening/intergenic noncoding RNAs (lincRNAs) have been linked to *O*-glycosylation involved in CRC progression [151]. Furthermore, it has been proposed that hypoxia-upregulated transcribed-ultra conserved regions (T-UCR), named hypoxia-induced noncoding ultra-conserved transcript (HINCUT), are critical for the optimal *O*-GlcNAcylation of proteins in oxygen deprivation in cancer [152]. Finally, recent mass spectrometry studies recognized glycolipids as potential biomarkers for various physiological and pathological processes, being involved in the development of neurological and neurodegenerative diseases, including Parkinson’s disease (PD), Alzheimer’s disease (AD), Lewy body and frontotemporal dementia [153].

#### 5.1.1. *N*-Linked Glycosylation

*N*-linked glycosylation is a very prevalent form of glycosylation in nature, being required for the proper folding of some eukaryotic proteins in the ER. Thus, a fourteen carbohydrate-long common oligosaccharide precursor (2 *N*-acetylglucosamine, 9 mannose and 3 glucose) is linked to the asparagine of the core nascent protein in the ER. This fourteen-carbohydrate common precursor is classified into three types, based on residues linked to the (Man)3(GlcNAc)2-Asn-peptide core. (1) “oligomannose”, consisting solely of mannose residues; (2) “complex”, constituted by “antennae” initiated by *N*-acetylglucosaminyltransferases (GlcNAcTs); (3) “hybrid”, consisting of mannose residues attached to the Manα1–6 arm of the core and one or two antennae to the Manα1–3 arm [47]. Once transferred to the nascent peptide chain, *N*-glycans undergo an extensive process, resulting in the removal of three glucose residues, as well as several mannose residues, depending on the *N*-linked glycan in elaboration. *N*-linked glycans are extremely important in proper protein folding in eukaryotes. The removal of these residues depends on the correct protein folding that occurs for translocation to the Golgi apparatus. Here, mannose residues can be removed and replaced by other monosaccharides (e.g., *N*-acetylglucosamine, *N*-acetylgalactosamine, galactose, fucose and sialic acid) to elongate the *N*-linked oligosaccharides [154]. Conversely, a mannose-6-phosphate sequence serves as a signal to move towards the lysosome, the unfolded protein to which this glycan is attached. This recognition pattern is allowed by the activation of two specific endocytic receptors for the glycanic sequences, the cation-independent mannose-6-phosphate receptor (CI-MPR) and the cation-dependent mannose-6-phosphate receptor (CD-MPR) [155]. Moreover, the clearance of secreted glycoproteins can also depend on the sialic acid. In fact, the loss of sialic acid from glycoproteins triggers clearance by the Kupffer cells carrying receptors for asialoglycoproteins [156]. Finally, *N*-linked glycans also play an important role in cell–cell interactions. This is the case of the CD337 receptor on Natural Killer cells indicating the recognized cell as cancerous [157].

#### 5.1.2. *O*-Linked Glycosylation

It is known that *O*-linked glycans are mainly catalyzed in the Golgi complex [158]. Here, the C-1 of *N*-acetylgalactosamine is covalently bonded to the hydroxyl of serine or threonine of the nascent core protein [158,159]. Once the N-acetylgalactosamine residue has been added, other carbohydrate residues such as galactose, fucose, *N*-acetylglucosamine and sialic acid can be added [160,161]. Thus, different types of *O*-linked oligosaccharides have been identified, including *O*-fucose, *O*-mannose, *O*-glucose and *O-N*-acetylglucosamine. The resulting structures play a role in modulating protein activity via different mechanisms: (1) phosphorylation, (2) protein–protein interactions, (3) protein degradation, (4) protein localization and (5) transcription [162,163,164]. Regarding the phosphorylation, many studies have found sites of attachment for *O*-phosphate and *O*-GlcNAc to be mapped to the same residue [163,164]. These data suggest that O-phosphate and *O*-GlcNAc modify proteins by competing for the same serine or threonine residues. Therefore, by acting on the availability of the latter residues, *O*-GlcNAc regulates protein function resulting from the phosphorylation patterns [163]. This is a biological topic, as an altered phosphorylation pattern can change enzymatic cascade and signaling pathways with consequent modification in expression level of genes encoding, for example, glycosyltransferases and glycosidases whose ration generates, in turn, the modulatory glycocode. In fact, covalent modification of proteins and enzymes through phosphorylation/dephosphorylation constitutes a sophisticated control system in cell homeostasis [165]. Moreover, proteins coupled to *O*-GlcNAc are efficiently shuttled from the cytoplasm to the nucleus in Aplysia neurons, suggesting a functional role of *O*-GlcNAc as alternative nuclear localizing or cytosol retention signal [163,164].

However, *O*-glycans are thought to work in general on the outer cell surface, through mucins, sLeX and selectins [26]. Specifically, unlike mucins that are GalNAc-linked, the addition of GlcNAc does not typically occur in the Golgi apparatus and is not extended [166]. This synthesis is regulated through *O*-GlcNAc-transferases (OGTs) and *O*-GlcNAcases (OGAs) [167]. These enzymes and their stoichiometric ratio exist depending on the subcellular compartment as well as gene expression. They perform a rapid cycle of addition and removal of GlcNAc from protein substrates. This dynamic process seems to be unique to this glycosylation motif also regulating metabolism and other cellular functions.

sLeX are important in ABO blood antigen determination and immune response. P-selectin released from Weibel–Palade bodies on blood vessel endothelial cells are involved in the contact mechanism between endothelial cells and bacterial peptidoglycan as well as lymphomonocytes or neutrophils during inflammation [166]. P-selectins can bind to the plasmalemmal sLeX on the neutrophils in the bloodstream, helping the extravasation of these cells during infection [168], but also the mononuclear cells during tethering and rolling over the BBB in the early phase of neuroinflammation detected in MS and related disorders [169].

Other significant *O*-linked glycoproteins are glycophorin of the erythrocyte cell membranes; notch, the transmembrane receptor involved in development and cell fate; thrombospondin; coagulation factor VII and IX; and the urinary type plasminogen activator. GAGs are polysaccharide polymers classified into four groups, based on the disaccharide core; they generally work in the ECM [170]. Heparin/heparan sulfate (HSGAGs) and chondroitin sulfate/dermatan sulfate (CSGAGs) are synthesized in the Golgi apparatus, where protein cores made in the rough endoplasmic reticulum are post-translationally modified with *O*-linked glycosylation by glycosyltransferases forming proteoglycans [171,172,173]. Keratan sulfate can have a *N*- or *O*-linked glycosylation core of the proteoglycan [174]. Finally, the hyaluronic acid is synthesized by integral membrane synthases [175]. The latter secrete the disaccharide chain that undergoes dynamic elongation. Therefore, GAGs are represented by heparin, heparan sulfate, chondroitin, keratan and dermatan. Some of these, such as heparan sulfate, are bound to the plasmalemma through a tetrasaccharide linked to a protein via a xylosyl residue [170].

#### 5.1.3. *C*-Linked Glycosylation

*C*-linked glycosylation or *C*-mannosylation concerns only 18% of human proteins because the sugar is linked to a carbon atom, a species that is less reactive than nitrogen or oxygen [176]. Only in 2011 was the first *C*-mannosylated protein determined using crystallography, the human complement component 8 [177]. This type of glycosylation implies that a mannose molecule is added to the first tryptophan residue in the sequence W–X–X–W, where W indicates tryptophan and X is the amino acid [178]. Thus, a C–C bond can be formed between the first carbon of alpha-mannose and the second one of tryptophan [178]. However, for reasons of binding energy, this is true only for two thirds of the cases because the second amino acid is preferred if belonging to the polar ones (Ser, Ala, Gly and Thr), for mannosylation to occur. Recently, the technique for predicting whether or not the sequence expresses a mannosylation site has advanced, providing an accuracy of 93% [176]. Thrombospondins are one of the main *C*-mannosylated proteins, as well as Type I cytokine receptors [179]. Numerous studies have shown that *C*-mannosylation is an important process in the secretion of proteins which are retained in the ER, if they do not undergo this type of glycosylation [176]. This also holds true for erythropoietin [180].

#### 5.1.4. Glypiation

Glypiation consists of the formation of GPI anchors that bind proteins to lipids through glycan linkages [181]. This special form of glycosylation frequently concerns ER enzymes involved in the maturation process of proteins [181].

#### 5.1.5. Phosphoglycosylation

Phosphoglycosylation is a rare alternative glycosylation type involving a number of newly identified glycoproteins containing oligosaccharides linked to serine or threonine in a peptide backbone via phosphodiesters [182]. This refers to xylose in Trypanosoma cruzi [183]. Meanwhile, mannose has been reported in Leishmania Mexicana [184], in mice and especially in Mus musculus on the cell-surface laminin receptor alpha dystroglycan [185]. Given the highly conserved form of alpha dystroglycan from vertebrates to mammals, the severe human dystrophies induced by its mutations are not surprising [186].

### 5.2. The Non-Enzymatic Glycosylation

Non-enzymatic protein glycosylation (glycation) is a common spontaneous post-translational in vivo modification of proteins, resulting from covalent attachment reactions between carbonyl group of a reducing sugar (mainly glucose or fructose) and the amino groups of peptide chains. These reactions take place across, or close to, water channels and protruding tubules not needing enzymatic intervention, and alter the structure and activity of the involved proteins, resulting in permanent residues known as advanced glycation end products (AGEs). The formation of AGEs (the Maillard reaction) starts with the reaction of sugar aldehydes with the *N*-terminus of free-amino groups of proteins to form a so-called Schiff base [187]. Rearrangements of the instable Schiff base lead to the formation of Amadori products. A small subset of Amadori products will undergo further irreversible reactions with oxidation, reduction, dehydration, condensation, fragmentation and cyclization leading to the formation of AGEs [188]. Currently, the incubation of proteins with lipid peroxidation products is an alternative method of generating AGEs as well as the polyol pathway [189]. The latter promotes the conversion of glucose into fructose; fructose may further be converted into 3-deoxyglucose and fructose-3-phosphate, both of which are very potent non-enzymatic glycation agents.

In the first classification system, the most extensively studied AGEs are *N*-carboxymethyllysine (CML), pentosidine, crossline, pyrraline and hydroimidazolone [190]. The second group includes AGE-1 (glucose-derived AGEs), AGE-2 (glyceraldehyde-derived AGEs), AGE-3 (glycolaldehyde-derived AGEs), AGE-4 (GO/glyoxal, MGO/methylglyoxal-derived AGEs), AGE-5 (glyoxal-derived AGEs), AGE-6 (3-deoxyglucosone-derived AGEs) and acetaldehyde-derived AGEs (AA-AGEs) [191]. Specific modifications of proteins are also considered AGEs; for example, glycated haemoglobin (called HbA1c) is actually an Amadori product, not an AGE, albumin (the experimental bovine serum albumin (BSA)-AGE), eye crystallin, collagen type IV and others [192]. Notably, the main sources of these *N*-glycosylated proteins in serum are the B-cells (immunoglobulins) and the liver (albumin), but also macrophages (cytokines) and other cell types. Finally, defined glycotoxins AGE-2, AGE-3 and AA-AGE are referred to as toxic end products of advanced glycation (TAGE) [193]. There is also compelling evidence that non-TAGE molecules, such as CML, GO, MGO, pentosidine, pyrraline and crossline may be cytotoxic, with LD50 of CML calculated to be >5 mg/kg [194]. In fact, in the presence of MGO, the translocation of Nrf2 from the cytosol to the nucleus is inhibited, which results in a decreased expression of detoxifying enzymes such as heme oxygenase-1 [195]. Nrf2 belongs to the fumarate pathway of action. Although, AGEs are generated endogenously during degenerative diseases and aging, also becoming markers of their pathological processes. Cooking foods and smoking are associated with exogenous AGEs as well [196,197]. Thus, some of the exogenous AGEs are carcinogenic, for example acrylamide or heterocyclic amines [198]. Interestingly, the average human diet consists of ~75 mg AGEs per day, 10–30% (30–80% in another report) of which is systemically absorbed and about 30% in turn removed in the urine [199].

In all cases, AGEs act through multi-ligand plasmalemmal receptors for advanced glycation end-products (RAGE) inducing NADPH-oxidase activity, with activation of Nf-κB and increased iNOS expression as well as STAT3, HIF-1α, AP-1 and CREB. This molecular cascade leads to oxidative stress, promoting inflammatory processes with a final cytotoxicity [194]. These effects are attenuated by antioxidants, such as thioctic acid, *N*-acetylcysteine, and active ingredients contained in green tea; garlic; resveratrol; red wine; curcumin; cinnamic acid derivatives, such as ferulic acid and quercetin [200]. In addition, the aminoguanidine, a Maillard blocker, induces a positive effect in reducing AGE accumulation in tissues in experimental diabetes and preventing the age-related cardiac hypertrophy and arterial stiffening [201]. Nevertheless, its side effects make it very difficult in human chronic use. This is important as glycated proteins, especially large ones, are resistant to proteolytic enzymes, making it more difficult for them to be eliminated from the body.

Among RAGE ligands, there are also pro-inflammatory molecules including amyloid peptides, S100/calgranulin proteins, high mobility group box 1 proteins (HMGB1) and LPSs [202,203,204].

As for glycans in general, there are also problems in the measurement of the in vivo chemical species for AGEs. The use of advanced methods such as chromatographic, immunoenzymatic (ELISA) or fluorescent has improved our detecting sensitivity [192,205]. Thus, accumulated AGEs in the skin can currently be estimated using the non-invasive autofluorescence measurement [206]. The studies derived from these techniques evidence a net increased AGEs level in obesity [187], Diabetes Mellitus [207] and chronic inflammatory diseases, like Rheumatoid Arthritis [208], as well as in age-related diseases, such as cardiovascular, renal and neurodegenerative pathologies [193,209]. RAGE, GO and MGO are overexpressed particularly in chronic inflammatory diseases [191]. These pathologies are associated with the immune-mediated inflammation and cell activation, resulting in a switch towards glycolysis, high glycolytic rate and AGEs production. In fact, the activation of CNS-resident microglia and infiltrated macrophages can induce a metabolic switch, promoting glycolysis over oxidative phosphorylation [210,211]. In MS, these metabolic modifications refer to T-cells and monocytes, but also to astrocytes after uptake of myelin and oligodendrocytes [212]. The accumulation of AGEs in the plasma and CNS of MS patients can contribute to neuroinflammation and progression of this pathology. In Alzheimer’s disease, β-amyloid peptide depositions and neurofibrillary tangles are affected by glycation [213], which is also increased in the cerebral cortex, amygdala and substantia nigra of Parkinsonian patients [214].

## 6. Glycodrugs

Many existing biologically active compounds used for therapeutic purposes are glycosides coming from plants, animals and bacteria. A large number of these have been structurally modified, resulting in derivatives and other ex novo synthetized ones, according to the platform-based or the prevailing click-based chemical approach, respectively [215]. Currently, disposable biopharmaceuticals are glycosylated proteins including monoclonal antibodies (mAbs), fusion proteins, growth factors, cytokines, therapeutic enzymes, and hormones. Moreover, advances in molecular glycobiology have clarified the relationship between aglycone and glycoside activity, often making it possible to develop more active glycodrugs [215]. In fact, glycosylation affects the pharmacokinetics, pharmacodynamics, and immunogenicity of a therapeutic compound. It can influence pharmacokinetics also by protecting proteins from proteolytic degradation in vivo [216]. We know why partially glycosylated proteins have a shorter lifetime than fully glycosylated ones, due to the binding of galactose with hepatic asialoglycoprotein receptors expressed on hepatocytes, which promotes hepatic clearance of the partially glycosylated proteins [217]. The latter, in fact, unlike the glycosylated ones containing sialic acid, usually contain only a terminal galactose. Apart from these findings, the main event in the history of glycodrugs was the Iminosugars discovery in 1970 [218].

Iminosugars, where a nitrogen replaces the endocyclic oxygen atom in the hemiaminal ring system of structure, are another important class of carbohydrates with medicinal properties and are also common components of plants [219]. Regardless of their clinical uses, these molecules enhance our knowledge in understanding the signaling and metabolic functioning of glucose in the cell, as well as mechanisms of viral and cancer development through pleiotropic effects deriving from inhibition of glucosidases. Other classes of glycodrugs act by interfering on cell compounds synthesis, protein–protein interaction or recognition pattern. This is the case of mAbs. In fact, IgG Fc glycosylation is critical to many functions of antibody effectors through modulating Fc-FcγR interactions [220]. The human FcγR family includes activating (FcγRIa, FcγRIIa, and FcγRIIIa) and inhibitory (FcγRIIb) receptors. Fc glycosylation plays important roles in modulating the antibody binding affinities with FcγRs or C1q on effector cells, and thus affects immune effector functions such as antibody-dependent cell-mediated cytotoxicity (ADCC), antibody-dependent cellular phagocytosis (ADCP), and complement-dependent cytotoxicity (CDC) [221]. These notions have been fundamental to understanding the mechanisms of action (MOA) of therapeutic antibodies and their clinical use. A quick excursus of the most representative drug molecules is described below, with no claims to completeness given the vastness of this matter.

### 6.1. The α-Glycosidases Inhibitors: Iminosugars

Suspended in water-soluble fractions of plants and microbial broths, iminosugars seem likely to be synthetized to protect carbohydrates produced during photosynthesis and to reduce competition from other microorganisms by inhibiting their glycosidases [222]. However, many of these natural products common in plants, bacteria and fungi are not inhibitors of any glycosidase. This suggests that iminosugars have other functions, all supported by the chaperoning activity, resulting in regulatory roles and immunomodulation in mammals. Indeed, iminosugars do not need to be glycosidase inhibitors for pharmacological activity, but a lack of glycosidase inhibition removes many off-target activities [223].

The first isolated iminosugar was the 1-deoxynojirimycin (DNJ). In 1976, it was found in the mulberry tree [224] as a biochemical activity, that is, a glycomimetic α-glucosidase inhibitor and for medical application as an anti-diabetic, anti-viral and anti-cancer agent progenitor. After that, the analog 1,4-dideoxy-1,4-imino-d-arabinitol (DAB) and others iminosugars (about 200) were also discovered as glycomimetics, representing a new generation of carbohydrate-based drug candidates for treatment of diabetes, viral infections including influenza, HIV, hepatitis C and B, as well as Dengue and cancer [225]. Specifically, DNJ induces both an anti-diabetic and a broad-spectrum anti-viral effect by interfering with protein-folding machinery, complex glucosides hydrolysis and food adsorption by inhibiting GANAB [218]. Protein-folding machinery, in fact, is exploited by most viruses to assemble capsid structural compounds after cell infection. Furthermore, the anti-cancer effect is due to the GANAB inhibition, affecting enhanced glycoprotein turnover, which is reflected by an extremely active lysosomal system and membrane trafficking in tumors [226]. However, due to abdominal pain and other adverse effects, DNJ never entered into the clinical routine but was modified to produce therapeutic derivatives [223,227]. Apart from iminosugars, ER stress and unfolded protein response (UPR) have been studied in human pathology, representing an emerging field as well as a fascinating aspect of diseases. In fact, the pathologic feature of many neurodegenerative diseases is the accumulation of misfolded proteins in the form of aggregates within affected neurons, although vascular and metabolic conditions are also thought to be involved [228,229].

### 6.2. Glycodrugs in Diabetes Mellitus and Thesaurismosis

Acarbose is a pseudo-tetrasacharide derived from cultures of Actinoplanes strain SE 50, which possesses a nitrogen molecule between the first and second glucose molecules. This modification confers a particularly high affinity for the α-glucosidase enzyme inhibition, resulting in a clinically relevant anti-diabetic effect [230,231].

*N*-hydroxyethyl-DNJ (Miglitol) [232] for diabetes and *N*-butyl-DNJ (Miglustat) [233] for Gaucher’s (in the USA) and Niemann–Pick type C disease (in Europe) have been synthetized and authorized for medical use, with the trade name of Glyset and Zaveska respectively. In particular, Miglitol establishes enhanced glycemic control by inhibiting the membrane-bond α-glucosidases of the small intestine and pancreas to hydrolyze carbohydrates into simpler absorbable forms. Since 1996, it has been approved for non-insulin-dependent diabetes mellitus, in which it reduces the complex carbohydrate digestion with a consequent decrease in glucose absorption and hyperglycemia.

Pyrrolidine iminosugar DAB, another potent inhibitor of α-glucosidases, has been shown to reduce glucagon-induced and spontaneous hyperglycemia in rats and dogs [225]. DAB-induced inhibition of hepatic glycogen phosphorylase improves glycemic control in patients with Type 2 diabetes [231].

Interestingly, DNJ and Miglitol were found to be potent agonists of the human glucose sensor, sodium/glucose cotransporter type 3 [234]. Miglustat inhibits the glucosylceramide synthase enzyme, which reduces biosynthesis of glucosylceramide from ceramide, resulting in reduced glycosphingolipids (GSLs) synthesis and deposition [235]. It also inhibits α-glucosidase I and II, lysosomal and non-lysosomal glucocerebrosidases, sucrase and maltase [235]. This decreases the excessive cellular storage of glycolipids in neural tissue, because Gaucher’s and Niemann–Pick type C are diseases caused by a deficiency in glucocerebrosidase and a deficiency in the metabolism of cholesterol and other lipids respectively [218].

Miglustat hydrochloride, (Amigal^®®^) is a pharmacological chaperone that selectively binds α-galactosidase A (α-Gal A), increasing physical stability, lysosomal trafficking, and cellular activity [225]. Disfunction of this enzyme causes the Fabry disease [236]. Importantly, the chaperoning activity of Amigal^®®^ is observed at concentrations that do not inhibit the α-Gal A or other galactosidases [236]. However, it works as a therapeutic agent.

A promising therapy for Tay–Sachs and Sandhoff diseases involves the use of β-*N*-acetylhexosaminidase inhibitors such as 2-acetamido-1,4-imino-1,2,4-tride-oxy-l-arabinitol (LABNAc) as a chemical chaperone to enhance the enzyme activity above subcritical levels in order to avoid glycolipid storage [237]. This agent represents an emerging therapeutic tool.

### 6.3. Viral Infections and Glycodrugs

Regarding the antiviral activity of DNJ, DAB and derivatives, interesting inhibition properties have been found in vitro concerning viral replication and capsid assembly, particularly in flaviviruses [238]. However, the in vivo evidence was disappointing. *N*-butyl-DNJ and Celgosivir (6-*O*-butanoyl- castanospermine) have been shown to inhibit HIV infectivity in vitro by blocking viral envelope glycoprotein trimming [239,240]. However, in both cases, the clinical development was problematic due to the compound’s toxicity profile and competition from other less toxic anti-HIV drugs. Celgosivir has also been shown to have a 30-fold greater antiviral activity than the parent compound against cytomegalovirus, influenza and HCV [225,241]. Unfortunately, this agent and the promising castanospermine also inhibit intestinal glycosidases and cause osmotic diarrhea [225]. Finally, *N*-Nonyl-deoxynojirimycin has a further glycosidase-independent antiviral mode of action against HCV by inhibiting the formation of the p7 ion channel, which is able to perform cation selective ion channels in planar lipid bilayers [242]. Viral neuraminidases are glycosidases (sialidases precisely) that hydrolyze the neuraminic acid present in animal tissue and bacteria during the infectious phase when the receptor bridging to the host cell takes place by elimination of the steric hindrance induced by the surrounding sialic acid on the glycocalyx [243]. The neuraminic acid glycomimetic Neu5Ac2en, other than iminosugar, inhibits sialidases by altering transition state analogue, resulting in neuraminidase inhibitor, thus working in treatment and prophylaxis of influenza A and B [244]. The FDA-approved drug is called Relenza.

### 6.4. Carbohydrate-Based Antibiotics

Several types of carbohydrate-based antibiotic are known, most of which are bacterial and fungal products. They generally affect the bacterial protein synthesis.

The first type contains molecules in which carbohydrates are linked to cyclitols or aminocyclitols, known as aminoglycosides such as streptomycin, gentamycin, kanamycin, amikacin and neomycin [245]. Aminoglycosides irreversibly bind to 30S ribosomal proteins and macrolides block peptide elongation by reversibly binding to the 50S ribosomal unit [246]. In the second type, carbohydrates are linked to nucleotide moieties such as liposidomycin, tunicamycin, mureidomycins, and so on [245]. Nucleosides have an inhibitory effect over phospho-MurNAc-pentapeptide translocase, resulting in a biosynthesis block of the peptidoglycan layer [247]. Currently, ramoplanin is the only clinically used antibiotic of this class. It is known to inhibit the *O*-GlcNAc transferase gene [245]. In the third type, carbohydrates can be linked to a macrocyclic lactone ring, constituting macrolide antibiotics such as azithromycin and erythromycin A [246]. The latter inhibits protein synthesis by binding to the peptidyl transferase site of the 50S ribosomal subunit, thus resulting particularly effective on Gram+. Its derivative compound is cethromycin.

### 6.5. Carbohydrate-Based Cancer Drugs

Swainsonine (mannose analogue) and castanospermine (glucose analogue) are iminosugar alkaloid glycosidases inhibitors, which also express anticancer properties and act on the protein-folding machinery [248]. These compounds show cytotoxicity and the inhibition effect on cancer cell metastasis, decreasing the toxicity of chemotherapics, also acting as immunomodulators [249,250]. However, none of these compounds have currently entered into the oncological clinical routine.

Cancer is associated with a profound modification of cell glycobiology. The enhanced expression of various glycosyltransferase enzymes such as *N*-acetylglucosaminyltransferase V (GalNAc-TV, GnT-V, MGAT5) are responsible for the increased number of *N*-glycans in tumors [251]. These alterations, in turn, are considered the hallmark for cancer progression. Despite the global scientific effort in drug development against cancer, most of the molecules are still under investigation. However, the studies on the possible approaches to this issue are very illustrative for the biology of glycans and tumors. For example, many groups have attempted to use known tumor-associated carbohydrate antigens (TACAs), rather than isolate novel ones for anti-cancer vaccine intervention [252]. The NIH Institute, in fact, has defined TACAs as important prognostic biomarkers [252]. Their glycolipid-based classification comprehends the gangliosides GM2, GD2, GD3, fucosyl-GM1, Globo-H, and Lewis Y (LeY) [253]. In particular, syalo mono-gangliosides (glycosphingolipids) GM2, GD2, and GD3 are involved in human melanomas and LeY, such as Sialyl Lewis A (sLeA), sLeX, sLeX-LeX, are human tumor-associated antigens [253]. Unfortunately, TACAs alone proved to be poorly immunogenic to induce an adequate anti-cancer T-cell dependent immune response. Consequently, researchers began to conjugate TACAs with T-cell stimulating protein carriers, including keyhole limpet haemocyanin (KLH), tetanus toxoid (TT), BSA, and diphtheria toxin (CRM197) [254]. These complexes revealed a self-immunogenic activity resulting in antigen-specific immunogenicity suppression. Thus, TACAs have been coupled with different compounds, including zwitterionic polysaccharide A1 (PSA1), Toll-like receptor 2 (TLR2) ligand, Pam3CysSerK4, and T-cell peptide epitopes, to develop self-adjuvant multi-component cancer vaccines [255]. In these cases, the carbohydrate compound confers the specificity of the immune response against tumor cells as well as the immunogenicity of the vaccine itself. Some of these have shown concrete results in undergoing clinical trials.

Thus, GD3 ganglioside vaccines and anti-idiotypic monoclonal antibodies, which mimic GD3 gangliosides, were carried out on melanoma patients with evidence of a low survival outcome [256]. Currently, some experimental vaccine therapeutics are approved, including GM2 KLH/QS-21 and MGV (GM2/GD2 KLH QS21) for malignant melanomas [257]; Theratope (sialyl-Tn Ag) for breast cancer [258]; IGN 301 (anti-idiotypic antibody) for LeY antigen associated with small cell lung cancer [259]. Moreover, the National Cancer Institute has declared MUC1 as a priority cancer antigen. MUC1 is a transmembrane protein overexpressed in various tumors (such as lung, breast, pancreas, kidney, ovary, and colon tumors) aberrantly and differentially glycosylated in cancer cells as compared to normal cells. Due to these distinguishable features, many research groups are now attempting to develop a vaccine compound from it [260].

Another adopted strategy for carbohydrate-based cancer drug discovery refers to increasing the number of glucose transporters (GLUTs) and lectins on the membrane surface as well as increasing the uptake of glucose by cancer cells at a rate higher than that of normal cells, referred to as the Warburg effect [17]. Several cytotoxic agents, including glufosfamide, chlorambucil, busulfan, docetaxel, paclitaxel, have been glycoconjugated to be less toxic to normal cells than parent aglycons [261]. These sugar prodrugs are thought to be cleaved by intracellular glycosidases, allowing the release of active drugs with improvement of their pharmacokinetic properties. However, more observations are required to validate the GLUT-mediated efficacy of these drugs. On the other hand, the radiolabeled glucose-analogue, 2-deoxy-2(18F)fluoro-d-glucose (18F-FDG), represents a diagnostic hallmark in tumors as cancer cells consume it, and it is detected in PET [19]. A recent overview by Smith and Bertozzi [262] has resumed the clinical impact of glycobiology in biomedicine research, according to the glycobiology-targeted therapeutics, including selectins, Siglecs and mammalian glycans. We report below only a few examples, but we encourage you to visit the aforementioned article to learn more. For instance, the authors included in the Pan-selectin antagonist group small molecules like Cylexin for ischaemia-reperfusion injury in infant heart surgery. The P-selectin antagonists group includes the Crizanlizumab for vaso-occlusive crisis in sickle cell disease and Inclacumab for myocardial infarction. Uproleselan (GlycoMimetics) for MM, Gemtuzumab for acute myeloid leukaemia, and Pinatuzumab for follicular lymphoma and diffuse large B cell lymphoma have been included in the E-selectin antagonists’ group; the CD33 antagonists group includes AL003 for Alzheimer’s disease. The Siglec-8 and Siglec-10 agonists groups include Lirentelimab for Keratoconjunctivitis and CD24Fc for immune-related adverse events associated with checkpoint inhibitors, respectively; while the group of Siglec-15 antagonists includes the NC318 for metastatic solid tumors. The MUC1 peptide plus poly-ICLC for lung carcinoma, the Trivalent (GM2/GD2/GD3–KLH) vaccine with OPT-821 for metastatic sarcoma, the GD2/GD3 lactone–KLH/OPT-821 vaccine for neuroblastoma have been included in the Mammalian glycan vaccines. The Anti-glycan antibodies group includes Oregovomab for ovarian cancer and Dinutuximab for neuroblastoma. Racotumomab for tumors with *N*-glycolylated gangliosides (neuroblastoma, Ewing’s sarcoma, Wilm’s tumour, retinoblastoma and glioma), Abagovomab for ovarian cancer have been included in the anti-idiotype antibodies. The CAR cell therapies include anti-GD2 CAR T and anti-GD2 CAR NKT for neuroblastoma.

Finally, the inhibition of specific glycosidases blocks the complete *N*-glycan processing, resulting in an anti-cancer effect. Some iminosugars such as swainsonine [263], deoxymannojirimycin [264], castanospermine [265] have proved to be good inhibitors. Swainsonine, in particular, inhibits lysosomal alpha1–3 and alpha1–6-mannosidase and also Golgi alpha-manosidase II. The inhibition of Golgi alpha-manosidase II by (-)-Swainsonine, in turn, can block the expression of the beta(1→6)-branched complex type *N*-glycans in malignant human and rodent cells. Thus, swainsonine hydrochloride (GD0039) underwent clinical evaluation, but neither disease progression nor toxicity were not affected in the phase I trial [263]. Although several carbohydrate agents have been synthesized and studied in clinical trials, the therapeutic outcomes are disappointing, and similar results come from commercially available carbohydrate-based therapeutic agents. In conclusion, more comprehensive studies are warranted based on the promising ways which have been explored so far.

### 6.6. Cardioactive Glycosides

Ouabain is a cardiac glycoside extracted from ripe seeds of *Strophanthus gratus* and bark of *Acokanthera ouabaio*, known in biological studies to inhibit Na^+^/K^+^ ATPase pomp in myocytes [215]. This results in an intracellular increase in sodium ions concentration that triggers intracellular Ca+ accumulation facilitating, in turn, the release of calcium ions by sarcoplasmic reticulum, with a final improvement of ionotropism and contractility [266]. Digoxin, instead, is a purified cardiac glycoside found in the foxglove plant *Digitalis lanata*, expressing same biological activity and is conventionally used for the treatment of atrial fibrillation and flutter [267].

### 6.7. Heparin

Heparin and its analogue heparin sulfate are well-known highly sulfated glycosaminoglycans found in the cell surface or extracellular protein matrix [171]. They modulate classical activity such as coagulation but also migration, differentiation, proliferation, and cancer metastasis. The anticoagulant activity is mediated by the activating antithrombin III (a serine protease inhibitor), which, in turn, blocks thrombin, thereby inhibiting blood coagulation factors, Xa and IIa [268]. However, heparin can also activate platelet factor 4 as well, causing serious side effects such as thrombocytopenia [268].

### 6.8. Carbohydrate-Based Vaccines

As for anticancer vaccines, carbohydrates also play a twofold key role for antimicrobial vaccines, providing the specificity of the immune response against pathogen and immunogenicity [269]. First in 1983, Pneumovax was marketed, constituted by a capsular polysaccharide. Subsequently, it was modified and presented as Pneumovax 23, containing isolated polysaccharides from 23 serotypes [270]. Other carbohydrate-based vaccines approved to date include the ActHiB, OmniHiB (Haemophilus b) for Influenzae type b; Typhoid Vi (Typhim Vi) for Typhoid fever; and Prevnar (pneumococcal conjugate Pneumonia caused by Streptococcus vaccine) for pneumonia [271].

### 6.9. Carbohydrate-Based α-Glucosidases

Benzyl 1,2,3-triazole derivatives, such as ribavirin, were found to inhibit the anti-HIV retroviral activity by 60–65%, at concentration of 50 μM, and then have been subjected to clinical evaluation [272]. This molecule belongs to nitrogen-containing heterocyclic compounds that are indispensable for life, being part of essential building blocks such as amino acids, nucleotides, etc.

1,2,3-Triazoles and derivatives are α-glucosidase inhibitors as well as one of the most important nitrogen-containing five-membered heterocycles, thus having many therapeutic applications ranging from antiviral, antitubercular and anticancer activity [273,274,275]. In particular, click chemistry compounds such as β-d-ribosyl, α-d-galactosyl, and α-d-xylosyl derivatives displayed maximum α-glucosidase inhibition and underwent assessment for putative clinical applications [276].

### 6.10. Glycodrugs Miscellanea

Dapagliflozin, a *C*-aryl glycoside, another iminosugar, was approved by the FDA in 2014 for glycemic control via sodium-dependent glucose cotransporter 2 (SGLT2) inhibition. Derivative fluorodapagliflozins have been synthesized by introducing a high-electron-withdrawing difluoro substituent, which decreases the negative charge of oxygen in the structure ring, resulting in better affinity with SGLT2 [277].

Topiramate, a sulfamate-substituted monosaccharide, has been approved by the FDA for the treatment of epilepsy, Lennox–Gastaut syndrome, and for the prevention of migraines [278]. It works by enhancing GABA (A) receptors, reducing membrane depolarization by AMPA/Kainate receptor activity, downregulating NMDA receptor activity, blocking voltage-gated sodium channels, inhibiting the glutamate one as well as the neuronal excitability as expected [279,280].

Vidarabine, an arabinosyl nucleoside analogue, first intended as anticancer drug, has been marketed as an antiviral drug against infections caused by herpes simplex and varicella zoster viruses [281]. After conversion to a monophosphate by viral thymidine kinase, this molecule is further modified to a triphosphate by host enzymes. Vidarabine triphosphate directly inhibits DNA polymerase, also acting as chain terminator in DNA replication [282].

Lactulose is a synthetic disaccharide (galactose and fructose) used against chronic constipation and hepatic encephalopathy [283]. In fact, it decreases the intestinal production and absorption of ammonia, while inducing an osmotic effect with evacuation [284].

Sucralfate, an aluminum hydroxide complex of sucrose sulfate, is used for the treatment of duodenal ulcers [285]. It dissociates in the acidic environment of the stomach to its anionic form, resulting in a protective barrier to pepsin and bile. This, in turn, inhibits the diffusion of gastric acid.

The orally administrated Auranofin, a carbohydrate-containing gold complex, is used as an antirheumatic agent [286]. Its main MOA is the inhibition of cellular redox enzymes, resulting in enhancement of oxidative stress and intrinsic apoptotic death.

## 7. Protein Folding and Folding Quality Control Machinery

The ER is a subcellular organelle where protein folding occurs. This phenomenon concerns a third of nascent proteins and 90% of all glycoproteins, working with an efficiency of 20%, albeit enhanced by several lumenal chaperones [287]. About 80% of these peptides entering the ER via the sec61αβγ translocon are immediately *N*-glycosylated, obtaining a Glc_3_Man_9_GlcNAc_2_ covalently asp-linked oligosaccharide [288]. In particular, Bat3, TRC35 and Ubl4A guide and facilitate the insertion of protein into the ER membrane with the aid of the Get1/2 receptor located on the ER-membrane [289]. Correctly folded proteins are sequentially deglycosylated and packaged into transport vesicles (part of the cell membranes trafficking system) and translocated to the Golgi complex according to the secretory pathway. The misfolded ones are retained within the ER lumen in a complex manner with calcium-dependent lectin-chaperone foldases Calnexin (CNX) and Calreticulin (CRT) [290]. These foldases are thought to be in a dynamic equilibrium with UDP-glucose glycoprotein glucosyltransferase (UGGT) that re-glucosilates the protein, restarting its folding process in the so called CNX/CRT cycle [290]. However, terminally misfolded/unfolded proteins (selected from the CNX/CRT cycle) bind to a critical HSP70 family member, BiP, undergoing ubiquitylation and further degradation via the 26S proteasome, according to the ERAD process, after retro-translocation to the cytosol mediated by Derlin-1 and Sec61 [291,292,293].

Accumulation of misfolded/unfolded proteins in the ER causes ER-stress and activates, in turn, the specific stress sensor signaling pathway, in the end, leading to the homeostatic (proteostatic) UPR [294,295]. The UPR modulation determines cell fate, depending on the quality and persistency of the foreign stressor. Consistently, during the adaptation phase, the slowed global translation reduces the ER folding load, while the upregulation of chaperons increases the degradation rate of unfolded proteins.

However, in the case of persisting pathogenic *noxa* and chronic inflammation, the proteostatic mechanisms of UPR become inadequate and stressed cells die due to apoptosis [296]. Several ER-stressors are known, including glucose deprivation, hypoxia, viral infection, point mutations promoting folding intermediates and their aggregation as well as aberrant calcium regulation, resulting in chaperone dysfunction [297,298]. Physiological processes such as aging can also influence the protein folding [299]. In summary, protein misfolding represents an emerging topic in biomedicine, due to its interesting pathophysiological implications in cancer [300,301,302,303,304], diabetes [305,306,307,308,309,310,311], neurodegenerative and chronic inflammatory diseases [312,313,314], but also therapeutic ones. In fact, the underlying molecular physiology is quite well-known, providing biological intervention and possible medications.

### The UPR

The molecular physiology of *N*-glycan-dependent quality control of glycoprotein folding prevents ER endoplasmic exit of misfolded forms and their aggregates. ER-resident GluI and GluII sequentially trim the three terminal glucose moieties on *N*-linked Glc_3_Man_9_GlcNAc_2_ glycan coupled *en bloc* to nascent glycoproteins by oligosaccharyltransferase (OST) [315,316,317,318]. First de-glycosidase reactions are essential for proper folding and cell proteostasis. In particular, the α-GluI operates a first cleavage by removing a glucose residue from the conserved Glc2Man9GlcNAc2 *N*-linked glycan attached to nascent glycoprotein, and the resulting Glc1Man9GlcNAc2 glycan allows it to bind the ER CNX/CRT and associated chaperone ERp57, belonging to the protein disulfide refolding isomerases (PDI) [319]. After the α-GluII–mediated second cleavage of the innermost glucose, the glycoprotein is linked to Man9GlcNAc2 glycan and loses binding affinity for the folding chaperones. Therefore, if properly folded, the glycoprotein can proceed toward the Golgi apparatus and secretion. If not, it undergoes UGGT-mediated re-glycosylation and recycling [320]. UGGT is a fascinating sensor for hydrophobic sequences, typical of unfolded proteins. Recycling enhances protein folding efficiency, avoiding ER loading. However, in the presence of high levels of unfolded/misfolded proteins in the ER, BiP dissociates from the *N*-terminus of ER stress transducers, leading to UPR activation [321]. When that occurs, a complex signaling cascade is activated through three ER transmembrane receptors: the activating transcription factor 6 (ATF6), the inositol requiring kinase 1 (IRE1) and the pancreatic endoplasmic reticulum kinase (PERK) [322,323]. These elements confer cell protection from stressors and metabolic adaptation. After BiP dissociation, PERK and IRE1 are activated through oligomerization and autophosphorylation generating p-PERK and p-IRE1, respectively. In turn, p-PERK phosphorylates eIF2α, forming p-eIF2α that reduces global protein synthesis and stimulates the translation of ATF4 [324]. The latter finally de-represses cytoprotective genes, autophagy-related genes, and ERAD-related genes. p-IRE1 splices XBP1 mRNA to generate the transcription factor sXBP that enhances chaperones and genes expression involved in ER expansion, ERAD, autophagy, and cytoprotection, also reducing ER load through a RIDD-dependent mRNA degradation [325]. ATF6α, once dissociated from BiP, transits to the Golgi complex where it is cleaved by the proteases S1P and S2P forming cATF6α [326]. The latter then migrates to the nucleus, where it stimulates chaperones, autophagy-related genes, ERAD-related genes, and cytoprotective genes. p-eIF2α levels are tightly regulated by the protein phosphatase 1 (PP1) and growth arrest and DNA damage 34 (GADD34) complex, which quickly dephosphorylates p-eIF2α, preventing detrimental long-term global protein biosynthesis inhibition [327]. Interestingly, GADD34 is upregulated by the CCAAT/enhancer binding (C/EBP) homologous protein (CHOP), a transcription factor whose expression is stimulated by ATF4; thus, the PERK-eIF2α pathway is regulated via a tight autofeedback loop [328]. Once CHOP is activated, BCL2 is targeted with final de-repression of caspases system and cytochrome c liberated by mitochondria, leading to apoptosis [329]. In vitro and in vivo studies have shown that activation of the PERK-eIF2α pathway leads to activation of NF-κB in oligodendrocytes and suggest neuroprotective effects of PERK signaling in MS and experimental allergic encephalitis (EAE), which is the animal model of MS [330].

Not by chance, PERK, GADD34, ATF6 α, eIF2α and CHOP are considered promising therapeutic targets for myelin disorders and other chronic inflammatory or degenerative organ specific diseases [328,331]. Moreover, PERK can influence the NRF2 signaling pathway because the latter is a direct substrate and effector of the PERK-dependent cell survival pattern [332]. In fact, during ER stress, once PERK has phosphorylated NRF2, it promotes its dissociation from Keap1 and the translocation to the nucleus where it binds to the antioxidant response element (ARE) for the transcription’s activation of genes encoding detoxifying enzymes [332]. These enzymes are the A1 and A2 subunits of glutathione S-transferase, NAD(P)H:quinone oxidoreductase, γ-glutamylcysteine synthetase, Heme oxygenase-1 (HO-1) and UDP-glucoronosyl transferase [333]. These data suggest PERK as a multiple-substrates phosphorylating agent, able to protect cell viability while avoiding oxidative stress. Coherently, Perk^−/−^ cells accumulate ROS if exposed to ER stress [334]. Notably, NRF2 is the main molecular target of BG12, a disease modifying treatment of relapsing remitting Multiple Sclerosis (RRMS) [335].

Regarding MS, although ER stress was postulated [336], no specific aggregation or misfolded protein was found, while in main neurodegenerative diseases such as PD, Huntington, AD, familial Amyotrophic Lateral Sclerosis (ALS) and Progressive Supranuclear Palsy (PSP), emerging evidence indicates pathogenic misfolding processes, UPR inducing protein package and well-known cell aggregates such as Lewy bodies (composed by α-synuclein), mutant huntingtin, neurofibrillary tangles (composed by τ- and phospho-τ-protein), mutant SOD1 and τ-tangles, respectively [337,338,339,340,341]. BiP and PERK are upregulated in PD, also expressing cell loss as a common feature of chronic neuroinflammatory and neurodegenerative conditions [342]. In fact, if UPR fails to restore proteostasis, cells initiate terminal programs such as autophagy or apoptosis [343].

Autophagy is an evolutionarily conserved cellular pathway in which a cell recycles its macromolecules and organelles starting from autophagosome. This is composed of a part of cytosol or cellular organelles, enclosed in a double membrane. It binds to endolysosomal vesicles, forming the autolysosome. The autolysosome is degraded, in turn, completing the cycle of autophagy. PERK, IRE1 and cytosolic Ca^2+^ are known to be autophagy effectors in ER stressed cells [343]. Programmed cell death is mediated by apoptosis, through the activation of aspartate-specific proteases, collectively known as caspases [344]. Stress-induced apoptosis can occur through both mitochondrial-dependent and independent pathways [345].

Although mitochondrial-independent pathways are not well understood, the mitochondrial-dependent pathway is, as it is induced by oligomerization of pro-apoptotic proteins, such as Bax and Bak [346]. Once Bax and Bak oligomerize, they move from the outer mitochondrial membrane, where they are sequestered in non-apoptotic conditions by the survival protein Bcl2, to fit into the mitochondrial membrane, breaching its integrity. This results in a net efflux of cytochrome-c from the mitochondria to the cytosol, initiating the Apaf-1 mediated caspase-9 activation pathway. Interestingly, Bcl2 is also localized in the ER-membrane [347]. The release of Ca^2+^ from the ER into the cytosol in response to ER stress facilitates this phenomenon, suggesting that this ion is key driver of membrane fission and caspase activation. In fact, ER is not simply a subcellular environment where reactions take place, but a stress-sensitive organelle that properly works on actively controlled redox and Ca^2+^ homeostasis in ATP-dependent manner, other than cytosol. Indeed, the ER-resident Flavoprotein Ero1p (ER oxidoreductin 1) is thought to be the oxidative factor serving as primary oxidase of PDI [348].

More molecular details of UPR have recently been added. Specifically, ER Hsp40 family proteins co-chaperones ERdj1 to 7 have been found [349]. These molecules play a critical role not only in stimulating ATP hydrolysis of BiP, but also in regulating its various activities [350]. Interestingly, dj1 is dysregulated in PD and upregulated in peripheral blood mononuclear cells (PBMCs) from MS [328,351].

Other protein is ERp44, which is located in the ER–Golgi intermediate compartment (ERGIC), and engaged in the folding/oligomerization or retention of some proteins [352]. Moreover, one of Selenoproteins is Sep15, which binds to UGGT and presumably works as a reductase [353]. Proteins that will be degraded if retained longer in the recycle process may raise the probability of mannose trimming of the polypeptide-bound *N*-glycans by ER-mannosidase I, a process known as the mannose timer model [354]. These mannose-trimmed structures are recognized by EDEM family proteins, belonging to ERAD, and they promote it [134].

Man_7_GlcNAc_2_ form of *N*-glycans generated after GluI and GluII activity is recognized by lectins OS9 and XTP3-B, which contain one and two mannose 6-phosphate receptor homology (MRH) domains, respectively [355].

Finally, unfolded regions of nonglycosylated proteins are recognized by ER chaperones, mainly by BiP, although the recognition mechanism is apparently distinct from that of glycosylated ones [350].

## 8. The GANAB

GANAB is the α-subunit of the glucosidase II heterodimeric enzyme, a member of the glycosyl hydrolase 31 (GH31) family of proteins. The prologue of its story begins in 1979 when the Glc_3_Man_9_GlcNAc_2_ lipid-linked precursor was isolated by Grinna and Robbins and assayed enzymatically in rat liver microsomes using phosphate buffer (50 mM, pH 6.75), and Triton X100 [317]. This dolichol-bound oligosaccharide is a highly conserved structure in eukaryotes, representing the specific substrate of GluI and GluII [356]. GluI removes the outermost α1,2-Glc, and then GluII trims second and third α1,3-Glc, by catalyzing hydrolyses at the Glc-α1,3-Glc and Glc-α1,3-Man glycosidic linkages, according to the following scheme:

$$\mathrm{Glc}\overset{1.2}{\to }\;\mathrm{Glc}\;\overset{1.3}{\to }\;\mathrm{Glc}\;\overset{1.3}{\to }\;\mathrm{Man}$$

Trimming two inner glucoses α1,3-Glc and α1,2-Glc from protein bound precursors produces, respectively, the Glc_2_Man_9_GlcNAc_2_-Asn as well as the Glc_1_Man_9_GlcNAc_2_-Asn and Man_9_GlcNAc_2_-Asn structures [357]. However, the kinetic reaction of these compounds is experimentally different, being immediate for the former, intermediate for the second, and slow for the third, reflecting, respectively, the step of entry in the maturation process, the linkage of chaperones, and finally the release of the latter, starting the secretory pathway of properly folded peptide [358]. Through these enzymatic assays, the authors contextually identified the α-(1,2)-glucosidase “O” as different from the α-(1,3)-glucosidase “AB”, confirming it in 1980 as the well-known heterodimeric enzyme α-glucosidase II [317].

### 8.1. The Structure and Localization Glucosidase II

α-Glucosidase II (GluII or GII) consists of a 110 kDA catalytic α-subunit (GIIα) and 60 kDA regulatory β-subunit (GIIβ). The latter contains a highly conserved terminal four amino acids, His-Asp-Glu-Leu (HDEL), noncatalytic domain and a lectin domain with homology to the MRH. It was first identified as a protein kinase C substrate 80 K-H (PRKCSH) by Hirai and Shimizu in 1990 [359] and determined as a part of the heterodimeric complex in 1996 by Trombetta and coworkers [360], finally as a Glc removing factor from protein precursor in 1999 by Herscovics and in 2002 by Trombetta and Parodi [361,362]. The co-expression of both subunits resulted in ER localization, suggesting that the active one is retained within the ER, due to the HDEL ER-retention signal present at the COOH-terminus of the regulatory subunit [363]. In fact, apart from the active site of the enzyme found in the β8α8 barrel domain, the domain primarily involved in binding GIIβ occupies the distal *C*-terminal side, interacting through the *N*-terminal one of GIIα-binding domain. In particular, two non-overlapping interaction domains, ID1 and ID2, have been identified [364]. ID1 consists of 118 amino acids at the NH2-terminus of the coding region and includes the cysteine-rich element region of GIIα. ID2 comprises amino acids 273–400 located at the COOH-terminus of GIIβ including a stretch of acidic residues [364].

The human α-subunit of α-glucosidase II is 944 amino acids long, including an NH2-terminal hydrophobic signal sequence followed by a 912 amino acid (103 kDa) catalytic domain containing no transmembrane domains or known ER-retention motifs [360]. The α-subunit contains a GH31 consensus sequence domain (G/F)-(L/I/V/M)-W-X-D-M-N-E providing a nucleophile retaining enzyme mechanism [365,366]. The homologue sequence obtained in S. cerevisiae by gene disruption confirmed this subunit as the catalytic one [360]. Moreover, mutagenesis of the Asp or Glc residue in the last four amino acids, D_564_MNE_567_ conserved motif obtained in Sf9 cells, was found to eliminate GluII activity, suggesting that these residues contribute to catalysis [365].

The human β-subunit of a-glucosidase II is 528 amino acids in length and contains an NH2-terminal signal sequence followed by a hydrophilic one of 514 amino acids (~58 kDa). The mature protein contains two EF-Hand motifs (Ca^2+^ binding loops), an acidic stretch of consecutive glutamic acids, several cysteine residues at the NH2 and COOH terminals [360], MRH domain, and a COOH- terminal HDEL sequence for ER retention/retrieval [367].

**Figure 1:**
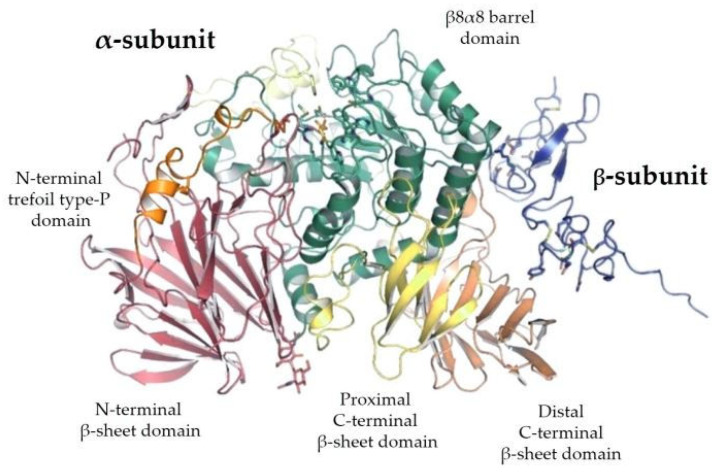
The ribbon structure of α-Glucosidase II heterodimeric complex. The α-subunit *N*-terminal trefoil type-P domain is shown in orange, and the *N*-terminal β-sheet domain is shown in pink. The α-subunit catalytic β8α8 barrel domain is shown in green. The Proximal and Distal α-subunit *C*-terminal domains are shown in yellow and light brown, respectively. Finally, the β-subunit is shown in blue.

The primary structure of GII presents overlapping sequences with several other glucosidases as well as MRH domain, but not with glucosidase I [368]. The MRH domain expresses a flattened, 9-stranded, β-barrel fold similar to other MRH domains, but the binding pocket was shallower than other homologous structures, probably due to the need to accommodate a single mannose residue. The MRH domain has four conserved residues (Gln-384, Arg-414, Glu-433, Tyr-439) essential for the binding of mannose and for the interaction with the terminal mannose on the 6′-pentamannosyl branch (*C*-branch) of the oligosaccharide substrate. Mutational analysis also revealed the functional role of Trp residue distal from the binding site, but it is essential for contacting the terminal a1,2-Man residue on the central branch of the Man9GlcNAc2 substrate, while the inner one exposes the Glc residue to the catalytic site on the GIIβ [369]. Figure 1 shows the GII 3D structure with functional domains.

**Figure 2:**
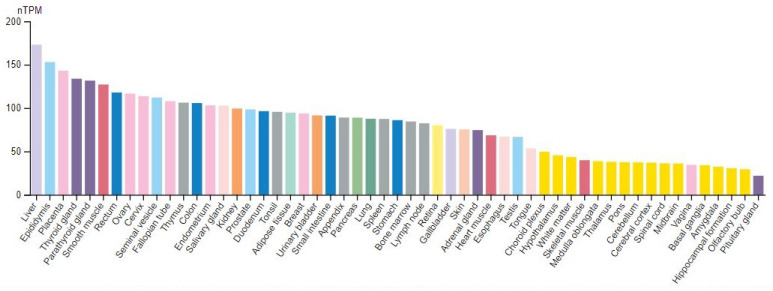
The human protein atlas of GANAB expression. nTPM normalized expression transcripts per million. (Figure reproduced from https://www.proteinatlas.org/ENSG00000089597-GANAB/tissue, accessed on 5 March 2022).

The main organ localization of GANAB is the liver, followed by the epididymis ductal cells as can be noted in Figure 2. As per the focus of our study, we can also find the kidney among the top sites of GANAB expression.

### 8.2. Enzyme Activity Assay and Interactions of Glucosidase II

GANAB and other GH31 neutral α-glycosidase C (GANC) are key enzymes in glycogen metabolism that hydrolyze terminal nonreducing 1,4-linked alpha-d-glucose residues from glycogen in the ER. GII activity can be assayed with several substrates. Specifically, natural substrates include Glc_1-2_Man_9_GlcNAc_2_ and maltose, but the enzyme does not hydrolyze glucose from Glc_3_Man_9_GlcNAc [370]. The enzyme pH optimum from various organisms ranges from 6 to 7.5 [371,372,373], but the human isoform coming from the human placenta has an even wider pH optimum of 5.5–8.5 [374].

Assays using p-nitrophenyl-a-D-glucopyranoside (pNP-Glc) as substrate (Km 0.85 mM for the rat liver enzyme) [372] provide reaction conditions containing 4 mM pNP-Glc, 50 mM-Hepes, pH 6.8, and 1% sodium cholate. Reactions are incubated at 37 °C and stopped with 0.5 M Na_2_CO_3_. Released p-nitrophenol is quantified through absorbance at 400 nm. A unit of enzyme activity is defined as the amount of enzyme that releases 1 mmol of p-nitrophenol/min [375]. Assays using 4-methylunbelliferyl-a-D-glucopyranoside (4-MU-Glc) as a substrate (Km 19 mM for the pig kidney enzyme) [371,373] provide reactions containing 0.1 mM 4-MU-Glc, 0.1 M citrate-phosphate buffer, pH 6.5. After incubation at 37 °C, reactions are stopped with 0.5 M glycine-NaOH, pH 10.4. Released 4-methylumbeliferone is quantitated using a fluorometer (excitation 360 nm, emission 450 nm). A unit of enzyme activity is defined as the amount of enzyme that releases 1 mmol of 4-methylumbeliferone/min [371].

Regarding interactions, unlike the UGGT that does not have an identified inhibitor (apart from its product, UDP) [319], GII inhibitors include the pyranose sugar analogues 1-deoxynojirimycin, *N*-5-carboxypentyl-dNM, and castanospermine, all iminosugars [218,376,377]. Other inhibitors have been identified in rat liver including p-chloromercuribenzenesulfonate (Ki 0.8 mM, IC50 0.55 mM), maltose (IC50 1.8–2 mM), glucose (IC50 17 mM), D-glucono-1,5-lactone (IC50 40 mM), Tris–HCl, and pH 6.6 (IC50 50 mM); a 10% glycerol solution reduces GII activity by 65 %. Moreover, 1% deoxycholate, 0.1% SDS, 1 M urea, and 0.5% iodoacetamide were found to irreversibly inactivate the enzyme [372]. Turanose (Ki 19 mM) was also found to inhibit enzymes from pig kidneys [373]. Finally, Bromoconduritol (6-bromo-3,4,5-trihydroxycyclohex-1-ene) inhibits the removal of the innermost α1,3-linked glucose residue from Glc_2_Man_9_GlcNAc_2_, leading to the accumulation of unprocessed high-mannose *N*-glycans in treated cells [378].

Regarding GII activators, 10 mM Man (172% activation), 50 mM Isomaltose (142% activation), and starch (7 mg/mL, 160% activation) activate the enzyme isolated from pig kidney microsomes [371].

The purification of the molecule for these assays was performed in rat liver microsomes using, in general, a combination of ammonium sulfate fractionation, anion exchange chromatography (DEAE cellulose and Mono Q), affinity chromatography (ConA Sepharose), gel filtration (Superdex 200), and hydroxyapatite chromatography providing enough purified enzyme to obtain peptide fragments for sequence analysis [360]. GIIα clones isolated from a human liver cDNA library encoded two distinct enzyme isoforms that differed in a 66 bp insertion, suggesting alternative splicing [363]. However, the co-expression of each isoform with the GIIβ in COS7 cells resulted in similar levels of enzyme activity [379].

Antibodies recognizing two different regions of the murine β-subunit were also generated [364].

Regarding gene deletion studies, a lectin-resistant mutant cell line from mouse lymphoma was found to be deficient in GII activity [380]. Cells accumulated glycoproteins containing Glc_2-1_Man_9-8_GlcNAc_2_ glycans, also expressing the complete loss of GII activity in vitro [381]. Moreover, Prkcsh-null mice used to study the development of autosomal dominant polycystic liver disease (ADPLD) evidenced that the gene deletion results in embryonic lethality [382]. Consistently, no multicellular organism is known to survive GII knockout to adulthood [383], and also homozygous UGGT deletion is embryonically lethal in mice [384]. In summary, mutations of GII or UGGT genes impair glycoprotein folding and cause ER retention and/or degradation with loss of function [10,385]. An in vitro association between CD45 (a transmembrane protein-tyrosine phosphatase, PTP) and α-glucosidase II has been reported in SAKR mouse T-lymphocyte cell line [386]. Indeed, CD45 is thought to be an essential regulator of T and B cell antigen receptor signaling, working by the activation of Src family kinases and suppression of JAK kinases [387]. Biologically, CD45 is known to be an enzymatic molecule, acting as a co-stimulator [388], but the role of its association with α-glucosidase II still remains unknown.

## 9. UPR in Human Diseases: The Role of GII

The involvement of GII in human physiology and pathology is evidenced through the enzyme inhibition effects by iminosugars as well as genetic mutations, such as Polycystic Liver Disease (PCLD) and Polycystic Kidney Disease (PCKD). In this way, we can obtain a concrete description of GANAB functions through its therapeutic inhibition, as in a loss-of-function experiment. Apart from iminosugar contribution, ER stress and UPR have been studied in human pathology, representing an emerging biological field and a fascinating physiopathological aspect of diseases themselves. Furthermore, except for MS and PCKLD, the relation between human pathology and α-glycosidases is only conceivable, based on the biological knowledge of ER stress, but has not yet been concretely demonstrated. In all cases, common aspects in the molecular physiology of UPR and ERAD suggest a therapeutic disease modifying approach, based on the inhibition of key mediators of apoptosis (such as CHOP, TXNIP, PERK and IRE1). This intervention has just been attempted in some cases.

### 9.1. Diabetes Mellitus

The glucostatic cycle is dysregulated in the diabetes mellitus, due to an insufficient number of β-cells that need to produce the required amount of insulin according to the fasted and postprandial states in order to maintain normoglycemia [389]. To support high-level insulin secretion, β-cells undergo highly developed ER stress [305]. Moreover, preproinsulin is co-translationally translocated in the ER lumen, where its signal sequence is clipped off and subsequently transformed into proinsulin through the formation of three intramolecular disulfides by ER-resident oxidoreductases intervention that allow it to fold to its native shape [390]. In the Akita mice model of disease, the mice suffer from insufficient insulin production secondary to β-cell loss, reaching toxic gain-of-function, ER stress with apoptotic pathway activation, and frank diabetes within 4 to 5 weeks after birth [308,391]. Rare infantile diabetes-causing Akita-like insulin mutations have been described in humans [392]. Interestingly, genetic removal of proapoptotic transcription factor CHOP, downstream of PERK, ameliorates β-cell loss and diabetes, emphasizing the central role of UPR in β-cell degeneration [308]. Coherently, in mice homozygous deletion of the gene encoding PERK causes massive and rapid β-cell apoptosis, leading to infantile diabetes. Interestingly, β-cells in Perk-null mice are distended with electron-dense proteinaceous material, also exhibiting a high rate of apoptosis [393,394].

### 9.2. Neurodegeneration

Accumulation of toxic protein species can kill neurons, and there is growing evidence that ER stress is an important mechanism driving this neurotoxicity [395]. In fact, the pathologic hallmark of many neurodegenerative diseases is already known as the accumulation of misfolded proteins in the form of aggregates within affected neurons [396]. We have previously referred to these topics, but regardless of the disease-specific aggregate, there is evidence of a kind of common final way leading to PERK hyperactivation in disease-affected brain regions [397]. Moreover, IRE1α activation and UPR induction are present in postmortem brain and spinal cord tissues in AD [398], PD [399] and ALS [400]. Additionally, spinal cord segments from autopsy of patients with sporadic ALS show ER stress resulting in the induction of UPR, chaperones, and apoptotic markers [400,401]. Up to prion pathology, brain samples from Creutzfeldt–Jakob disease show activation of a number of ER chaperones and other ER stress markers. Based on these data, drug intervention and UPR manipulation have been attempted in mouse models of prion-induced neurodegeneration. In fact, the oral administration of highly selective PERK inhibitors significantly reduces neurodegeneration and clinical disease in prion-infected mice, crossing the BBB efficiently [402,403].

### 9.3. Cancer

**Figure 3:**
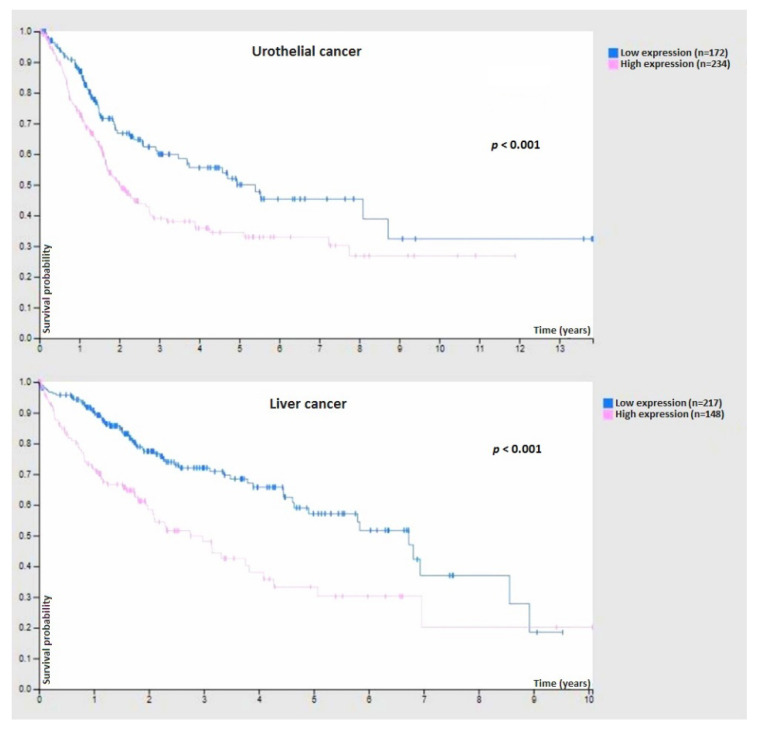
Prognostic relevance of GANAB expression in urothelial cancer (**top**) and liver cancer (**bottom**). In both cases, a high expression of this molecule is a marker of an unfavorable clinical outcome. (Figure reproduced from https://www.proteinatlas.org/ENSG00000089597-GANAB/pathology, accessed on 5 March 2022).

Tumor cells often invade or metastasize into foreign environments where unfavorable conditions, such as hypoxia, glucose deprivation, lactic acidosis, oxidative stress, and inadequate amino acid supply, compromise protein folding in the ER [404,405]. Many studies have found evidence of sustained and high-level activation of all three branches of the UPR (PERK, ATF6, IRE1α) in different tumors, including glioblastoma, MM, carcinomas of the breasts, stomach, esophagus, and liver [304,406,407,408,409]. It is currently unknown if ER stress is a *primum movens* of the transforming process or its effect. Anyway, in this topic, we have of a proteasome inhibitor, Bortezomib. This drug leads to myeloma cell death in part by preventing misfolded proteins through the ERAD pathway, thus triggering ER stress-induced apoptosis [410,411]. On this basis, inhibitors of the IRE1α RNase activity have recently been tested on human myeloma xenografts and their antimyeloma activity has been found [412]. On the other hand, the downregulation of XBP1s expression in myeloma correlates with resistance to Bortezomib [413,414], suggesting that the comprehension of UPR effects on tumors is more complicated than we thought. Despite the low tissue and cancer specificity of GANAB, the latter evidenced prognostic ability in liver and urothelial tumors. In fact, a statistically unfavorable clinical outcome is predicted in case of the high expression of this molecule in affected patients, as represented in Figure 3.

### 9.4. Ischemia-Reperfusion Injury and Atherosclerotic

Reduced blood flow as a result of arterial occlusion or hypotension causes tissue hypoxia and hypoglycemia [415]. These conditions rapidly induce protein misfolding and ER stress [416]. When blood flow is restored, reperfusion of affected tissues leads to oxidative stress and alterations in the redox state of the ER that disrupt the protein disulfide formation and cause ER protein misfolding [417,418].

Moreover, in the atherosclerotic plaques, there is evidence of UPR activation [419]. Moreover, high cholesterol levels, fatty acids, and oxidative stress can trigger ER stress-induced apoptosis of macrophages and endothelial cells associated with atherosclerosis [420]. Finally, brain regions affected by stroke also show ER stress-induced apoptosis [421].

### 9.5. ADPLD and ADPKD

Autosomal dominant polycystic kidney and liver disease (ADPKD and ADPLD, respectively) have been linked to pathogenic GII variants. These constitute recent phenotypes of congenital disorders of glycosylation (CDG). CDGs comprise all genetic defects associated with hyper- or hypo-glycosylation [422]. ADPKD and ADPLD are cases of the latter condition.

Unlike the mutations in GIIβ associated with ADPLD, the phenotypic picture of ones in GIIα is less clear, given its link to ADPKD [423,424].

In particular, all genes involved in ADPLD encode proteins involved in the ER trafficking and quality control of glycoproteins. The only exception is the LRP5, a transmembrane protein, part of the LRP5/LRP6/Frizzled co-receptor complex in the canonical Wnt signaling pathway [425].

In fact, although ADPLD and ADPKD are two distinct genetic disorders, they share PLD as a major phenotypic feature. Studies revealed that mutations in Prksch or Sec63 greatly reduce the expression, stability and proper trafficking of polycystin-1 (PC1), leading to cyst formation in a dose-dependent manner [426,427]. The level of functional PC1 at the cilium is thought to be central to the development of both hepatic and kidney cysts [428]. If the functional level of PC1 drops below a critical threshold, cyst development begins. At present, genes responsible for ADPLD are PRKCSH, SEC63, SEC61B, GANAB, ALG8, DNAJB11 and ALG9, which only explain 25–30% of the genetic spectrum of disease [429,430]. On the contrary, almost all ADPKD patients harbor gene mutations in polycystic kidney disease 1 (PKD1) or polycystic kidney disease 2 (PKD2) [431].

However, genetic mutations of GANAB are the only ones that have been shown to cause polycystic liver disease in patients affected by ADPLD or ADPKD as a kind of pathogenic continuum between these two forms. Treatment with proteasomal inhibitors increases levels of PC1 in cells by reversing the phenotype and providing a potential therapeutic approach [382].

This finding demonstrates the role of GIIα and GIIβ as fundamental in cyst development, due to protein folding dysregulation. A first MOA in the common feature of all truncating mutations in 80K-H/GIIβ is the loss of the HDEL [432]. Without the latter, enzymatic retention in the ER through HDEL interaction with the Lys-Asp-Glu-Leu (KDEL) receptor cannot take place, and the molecule would enter the secretory pathway and be trafficked out of the cell. Currently, similar to PRKCSH and SEC63, also other genes that encode proteins and belong to the protein biogenesis pathway in the ER are known, suggesting a unifier mechanistic hypothesis on cystogenesis. Lipid-linked oligosaccharide precursors of *N*-linked glycans are initially assembled in dolichol element on the ER membrane. After flipping it into the ER lumen, ALG8 catalyzes the addition of the second glucose residue [433]. Nascent polypeptides undergo a co-translational translocation via the SEC61 translocon pore composed of α, β and γ.

γ subunits and associated with SEC62. On the other hand, SEC63 and ERdj1 act in relation to chaperone BiP, facilitating this translocation process. Once OST catalyzes the attachment of the glycan moiety to asparagine residues, first, GI removes the outermost glucose residue, and then GII removes the second one, as expected [433]. This step causes the nascent protein to enter the folding CNX/CRT cycle. The subsequent removal of the innermost glucose by GII allows for the exit of the protein, if properly folded. In this case, it can proceed along the secretory pathway. On the contrary, misfolded proteins meet UGGT and once again are subjected to the folding cycle. Terminally unfolded proteins undergo ERAD and, through the SEC61 translocon complex, retrotranslocate into the cytoplasmic compartment, where they will encounter the proteasome. It is no wonder if PRKCSH, SEC63, SEC61B, GANAB, ALG8, DNAJB11, and ALG9 encode proteins that function in the post-translational ER protein biosynthetic pathways. However, mutations in SEC63 and PRKCSH are known to cause PCLD, also reducing directly the working dosage of PC1 in the bile duct and kidney tubules cells [433]. The PC1 loss of function can induce cystogenesis in a dose-dependent manner, once the lower threshold is crossed [434].

This continuum also applies to ADPKD, taking into account the lower tolerance in the PC1 cystogenic values of bile ducts compared to the kidney tubules one. In fact, a reduction in the steady-state levels of PC1 is documented in ADPLD-patients [382]. Notably, the loss of Sec61b, Sec63 and PRKCSH results in a severe deficiency of PC1; Alg8 knockout causes the hypoglycosylation of PC1; GANAB knockout results in a defective glucose trimming of *N*-glycan moieties, resulting in increased apparent molecular mass for PC1 [433]. LRP5 also seems to be involved in PC1 disfunction, having recently been recognized as a coreceptor for Wnt noncanonical signaling pathway [435]. Consistently, the silencing of each of these genes results in activation of at least the IRE1α/XBP1 branch of UPR [436]. From these observations derive the different abilities of PRKCSH and GANAB in activating the UPR, despite being subunits of the same GII holoenzyme. In fact, GANAB loss is a major determinant in inducing cellular severity compared to PRKCSH, suggesting the GII catalytic subunit’s lead role in ER stress.

### 9.6. Epididymal Pathology and Male Infertility

Few articles deal with GII activity and male infertility, particularly deriving from epididymal pathology, such as varicocele and epididymitis [437,438]. Although the exact role played byα-glucosidase in sperm function is not well understood, it can be speculated that the enzyme is responsible for sperm differentiation through protein modification and maturation [439]. In fact, GII activity is significantly reduced when an inflammatory occlusion distally to the epididymis occurs [440,441,442], in the case of functional deficiency of the latter in non-azoospermic patients [443], as well as a poor ability to bind the zona pellucida, thus suggesting a role of the enzyme also in sperm–egg binding [444]. The latter finding is consistent with a fine glycosylation control requested for the acrosome reaction to work normally, also becoming critical in sperm differentiation where high protein trafficking and maturation takes place. Although it has not entered into the clinical routine, the determination of GII activity is currently considered a useful tool in the diagnosis of epididymal patency and sperm abnormality [445].

### 9.7. Systemic Lupus Erythematosus

Systemic lupus erythematosus (SLE) is an autoimmune disease associated with both genetic predisposition and environmental influences. In 2006, Deng and coworkers attempted to characterize gene expression of CD4+ T lymphocytes in patients suffering from this disease [446]. Genome-wide expression profiles revealed several upregulated genes, including GANAB at high levels in the active state of SLE. The authors stated that gene expressions in CD4+ T lymphocytes could increase apoptosis, resulting in excessive uploading of self-antigens and a worsening of the disease.

### 9.8. Multiple Sclerosis

Multiple Sclerosis (MS) is an autoimmune demyelinating disease of the CNS with an inflammatory and degenerative component, also representing the main cause of nontraumatic disability in the young. In 2009, De Masi and coworkers first evidenced GANAB as a differentially expressed protein from PBMCs in naive patients compared to the RRMS ones undergoing IFN therapy [447]. In particular, a Spearman rank test applied to a proteomic approach coupled with MALDI-TOF analysis and quantitative brain MRI segmentation evidenced a good correlation between GANAB, lesion load (LL) and disease duration (DD).

More recently, De Masi and Orlando, by using Western blotting from PBMCs and MRI post-analysis of the brain, confirmed these findings and also described a significant correlation between GANAB expression and the Rio score [11]. The Rio score is a gold-standard method to classify the risk of disease progression based on one-year clinical/paraclinical observations in IFN-treated MS patients [448]. The molecule, in fact, resulted upregulated in RRMS untreated patients compared to the IFN-treated ones as well as the ones treated with disease modifying therapies (DMTs) other than IFN, including Dimethyl Fumarate (DMF, also called BG12). Moreover, GANAB was downregulated in treatment responder patients versus nonresponder ones, thus assuming higher expression values in relapsing patients than in clinically stable ones. Interestingly, levels of the glycozyme were higher in healthy controls (HCs) than in RRMS untreated patients, although in the latter group, the GANAB expression correlated with DD and LL values. This different expression between HCs and RRMS has been attributed to the well-known phenomenon of immunosenescence already described in MS [449,450]. Based on this evidence, the authors proposed GANAB as a biomolecular marker of neuroinflammation and treatment response in MS.

To our knowledge, no other published articles are available in the MS literature regarding GANAB, but several data indicate the improving effect of iminosugars on EAE [451,452,453].

## 10. Conclusions

During the evolution of the species, phylogenetically diverse organisms have used the extreme glycans variability to obtain control tools for cellular functions. This variability is due to the absence of a template, unlike proteins needing DNA for their synthesis. Despite this variability, the so-called “phenomenon of convergence” took place, resulting in a common glycocode development, specific for each basal cell function. The glycocode is determined by the concentration of glycosidase, glucosyltransferase and their substrates. The ratio of these enzymes is genetically regulated and post-translationally modulated, resulting in the internal and external cell recognition pattern, critical for cell viability and adaptation.

Although glycans are subject to micro- and macro-heterogeneity, some glycocodes have remained unchanged and highly conserved from yeast to mammals, including the *N*-glycan one for protein quality control (PQC). PQC is subjected to a fine regulation through key enzymes, including GII. The latter is linked to the ER membrane through its regulatory subunit, while the catalytic one is responsible for innermost glucose trimming on the Glc_3_Man_9_GlcNAc_2_ precursor signal. This reaction allows the nascent peptide to enter the CNX/CRT cycle for maturation.

The molecular checkpoint of this process is UGGT. UGGT acts in dynamic equilibrium with GII, according to the reglucosilation/deglucosilation activity, in order to allow access to the secretory pathway for properly folded proteins or cytoplasmic ERAD for the terminally unfolded ones. In the case of unfavorable environmental conditions or pathogenic noxa, cellular stress sensors are activated and UPR occurs. UPR is a secondary metabolic attempt to stress escape through PERK, IRE1 and ATF6 response by BiP de-repression, resulting in folding chaperone modulation and downregulation of protein synthesis for physiological adaptation. Long-acting stressors and chronically unresolved UPR induce unfolded protein loading and intracellular aggregates accumulation with final apoptosis by targeting CHOP and XBP1, leading, in turn, to organ pathology. Consistently, UPR and protein aggregates in target cells are documented in neurodegeneration, experimental diabetes, cancer and other chronic conditions as well.

Interestingly, emerging findings evidence UPR and related GANAB dysregulation or direct α-glycosidase involvement only in MS and SLE. Specifically, UPR can affect MS, resulting in PERK-related alteration of the Nrf2 signaling pathway. Notably, Nrf2, which belongs to the fumarate MOA, is the main molecular target of BG12 (DMF), a DMT of RRMS.

This small molecule is thought to affect disease progression and neuroinflammation by acting on the Hydroxycarboxylic Acid Receptor 2 (HCAR2) as well [454]. However, the PERK involvement suggests an additional MOA concerning ER stress in MS. The BG12-induced modulation of GANAB in PBMCs from MS patients was previously found by our group, corroborating this hypothesis. In addition, this finding is consistent with the alleviation of psoriasis vulgaris, also biologically characterized by UPR [455,456].

Evidence of the indirect involvement of GII comes from interventional studies concerning the effects of iminosugars on stressed cells in cancer, diabetes and viral infections. Moreover, genetically-induced loss of function in GII induces PCLD and PCKD, thus considered an *experimentum naturae* concerning the cystogenic effects of primitive ER impairment on protein trafficking in predisposed cells of bile ducts and renal tubules.

GANAB downregulation in response to DMT and its modular correlation with LL, DD and the Rio score in MS confirms the importance of this molecule in the physiopathology of the disease, reflecting neuroinflammation and suggesting MS as a misfolding pathology.

Similar considerations relate to diabetes, cancer and viral infections in which inhibition of GII by iminosugars attempts to rebalance the stressed system, resulting in improved glucostatic cycle, affected tumor invasion and capsid replication. All these findings indicate GII as key factor in physiology and human pathology, by acting on *N*-glycan substrate linked to nascent polypeptides in the ER.

## Data Availability

Not applicable.

## Abbreviations

**α** **-Gal A**	α-galactosidase A;
**18F-FDG**	glucose-analogue, 2-deoxy-2(18F)fluoro-D-glucose;
**4-MU-Glc**	4-methylunbelliferyl-a-D-glucopyranoside;
**AD**	Alzheimer’s disease;
**ADCC**	antibody-dependent cell-mediated cytotoxicity;
**ADCP**	antibody-dependent cellular phagocytosis;
**ADPKD**	Autosomal Dominant Polycystic Kidney Disease;
**ADPLD**	Autosomal Dominant Polycystic Liver Disease;
**AGEs**	advanced glycation end-products;
**ALS**	Amyotrophic Lateral Sclerosis;
**ARE**	antioxidant response element;
**Asn**	asparagine;
**ATF**	activating transcription factor;
**BBB**	blood brain barrier;
**BBE**	Bickerstaff’s brainstem encephalitis;
**BSA**	bovine serum albumin;
**C/EBP**	CCAAT/enhancer binding protein;
**CDC**	complement-dependent cytotoxicity;
**CDG**	Congenital Disorders of Glycosylation;
**CD-MPR**	cation-dependent mannose-6-phosphate receptor;
**CHOP**	C/EBP homologous protein;
**CI-MPR**	cation-independent mannose-6-phosphate receptor;
**CID**	Chronic inflammatory disease polyneuropathy;
**CML**	*N*-carboxymethyllysine;
**CNS**	central nervous system;
**CNX**	Calnexin;
**CRC**	colorectal cancer;
**CRM197**	diphtheria toxin;
**CRT**	Calreticulin;
**CSGAGs**	chondroitin sulfate/dermatan sulfate;
**DAB**	analog 1,4-dideoxy-1,4-imino-D-arabinitol;
**DAMPs**	damage-associated molecular patterns;
**DD**	disease duration;
**DMF**	Dimethyl Fumarate;
**DMT**	disease modifying therapy;
**DNJ**	1-deoxynojirimycin;
**EAE**	experimental allergic encephalitis;
**ER**	endoplasmic reticulum;
**ERAD**	ER-associated degradation;
**ERGIC**	ER-Golgi intermediate compartment;
**FAD**	flavin adenine inucleotide;
**GADD34**	growth arrest and DNA damage 34 complex;
**GAGs**	glycosaminoglycans;
**GANC**	GH31 neutral α-glycosidase C;
**GBS**	Guillain-Barré-Strohl syndrome;
**GH31**	glycosyl hydrolase 31;
**GIIα**	α-subunit of α-glucosidase II;
**GIIβ**	β-subunit of α-glucosidase II;
**GluI/GI**	α-glucosidases I;
**GluII/GII**	α-glucosidases II;
**GLUTs**	glucose transporters;
**GM-CSF**	granulocyte-macrophage colony-stimulating factor;
**GNS**	*N*-acetylglucosamine-6-sulfatase;
**GSD**	glycogen storage diseases;
**GSLs**	glycosphingolipids;
**HCAR2**	Hydroxycarboxylic Acid Receptor 2;
**HCs**	healthy controls;
**HDEL**	His-Asp-Glu-Leu;
**HINCUT**	noncoding ultra-conserved transcript;
**HMGB1**	high mobility group box 1 protein;
**HSGAGs**	heparin/heparan sulfate;
**IFN**	interferon;
**IRE1**	inositol requiring kinase 1;
**KDEL**	Lys-Asp-Glu-Leu;
**KLH**	keyhole limpet haemocyanin;
**LABNAc**	2-acetamido-1,4-imino-1,2,4-tride-oxy-l-arabinitol;
**LeY**	Lewis Y;
**LL**	lesion load;
**LPS**	lipopolysaccharide;
**MM**	Multiple Myeloma;
**MMP9**	matrix metalloproteinases type 9;
**MOA**	mechanisms of action;
**MOGAD**	Myelin oligodendrocyte glycoprotein antibody-associated disease;
**MRH**	mannose-6-phosphate receptor;
**MS**	Multiple Sclerosis;
**NAD**	nicotinamide adenine dinucleotide;
**Neu5Gc**	*N*-Glycolylneuraminic acid;
**NLRs**	NOD-like receptors;
**NMO**	Neuromyelitis Optica;
**NMOSD**	Neuromyelitis Optica Spectrum Disorders;
**OGA**	*O*-GlcNAcase;
**O-GlcNAc**	*O*-linked *N*-acetylglucosamine;
**OGT**	*O*-GlcNAc-transferase;
**OST**	oligosaccharyltransferase;
**PAMPs**	pathogen-associated molecular patterns;
**PBMCs**	peripheral blood mononuclear cells;
**PC1**	polycystin-1;
**PCKD**	Polycystic Kidney Disease;
**PCLD**	Polycystic Liver Disease;
**PD**	Parkinson’s disease;
**PDI**	protein disulfide refolding isomerases;
**PERK**	pancreatic endoplasmic reticulum kinase;
**PET**	positron emission tomography;
**PG**	peptidoglycan;
**pNP-Glc**	p-nitrophenyl-a-D-glucopyranoside;
**PP1**	protein phosphatase 1;
**PQC**	protein quality control;
**PRKCSH**	protein kinase C substrate 80 K-H;
**Pro**	proline;
**PRR**	pattern recognition receptors;
**PSA**	prostate specific antigen;
**PSA1**	zwitterionic polysaccharide A1;
**PSP**	Progressive Supranuclear Palsy;
**RAGE**	receptor for advanced glycation end-products;
**RRMS**	relapsing remitting Multiple Sclerosis;
**SAMPs**	self-associated molecular patterns;
**Ser**	serine;
**SGLT2**	sodium-dependent glucose cotransporter 2;
**SLE**	Systemic Lupus Erythematosus;
**sLeA**	sialyl Lewis A;
**sLeX**	sialyl Lewis X;
**TACAs**	tumor-associated carbohydrate antigens;
**TAGE**	toxic end-products of advanced glycation;
**TCR**	T-cell receptor;
**Thr**	threonine;
**TLRs**	Toll-like receptors;
**TT**	tetanus toxoid;
**T-UCR**	transcribed-ultra conserved regions;
**UGGT**	UDP-glucose glycoprotein glucosyltransferase;
**UPR**	unfolded protein response.
